# Global, Regional, and National Burden of Tuberculosis Among Children: A Population-Based Study

**DOI:** 10.3390/tropicalmed11020043

**Published:** 2026-02-05

**Authors:** Leiwen Fu, Ke Liu, Yuxian Sun, Wei Shu, Yujia Ning, Yang Liu, Jian Du, Liang Li

**Affiliations:** Beijing Chest Hospital, Beijing Tuberculosis and Thoracic Tumor Research Institute, Capital Medical University, District 1, No.9 Beiguan Street, Tongzhou District, Beijing 101149, China; fulw3@alumni.sysu.edu.cn (L.F.); lk9971@outlook.com (K.L.); yuxian960224@yeah.net (Y.S.); shuwei@tb123.org (W.S.); ningyujia@tb123.org (Y.N.)

**Keywords:** global burden of diseases (GBD), tuberculosis, children, incidence, DALY

## Abstract

**Background:** Tuberculosis remains a major global public health challenge, particularly among children. This study aims to provide a comprehensive assessment of the global, regional, and national burden of tuberculosis among children (0–14 years) using data from the Global Burden of Disease (GBD) 2021 study. **Methods:** Data on the incidence of tuberculosis (drug-susceptible, MDR-TB, and XDR-TB), as well as disability-adjusted life years (DALYs), among children aged 0–14 years in 204 countries and territories from 1990 to 2021 were obtained from the GBD 2021 study. Estimated annual percentage changes (EAPCs) in age-standardised incidence rates (ASIRs) and DALY rate were calculated overall and stratified by age, sex, and sociodemographic index (SDI) to quantify temporal trends. Spearman correlation analyses were performed to assess associations between tuberculosis burden and SDI. **Results:** In 2021, there were an estimated 759,300 new tuberculosis cases (ASIR: 37.7 per 100,000 population) among children globally, including 32,515 cases of MDR-TB (ASIR: 1.6) and 1193 cases of XDR-TB (ASIR: 0.1). Both global ASIR and DALY rate exhibited a declining trend from 1990 to 2021, with EAPC of −2.61% (95%CI: −2.74 to −2.47) and −4.38% (−4.61 to −4.14), respectively. From 1990 to 2021, High-income North America was the only GBD region with an increasing ASIR for tuberculosis (EAPC = 1.12, 95% CI: 0.61 to 1.64). From 1990 to 2021, there was no significant change in ASIR of MDR-TB (EAPC = 1.18, 95% CI: −0.16 to 2.54). However, eight of the 21 GBD regions exhibited increasing trends in the ASIR of MDR-TB, with the largest increase observed in Oceania (11.99, 10.49 to 13.52), followed by Central Asia (9.76, 6.48 to 13.13) and South Asia (5.71, 3.10 to 8.38). A strong negative correlation was observed between tuberculosis burden and SDI, with the highest disease burden concentrated in low-SDI regions. **Conclusions:** Achieving elimination targets will require stronger diagnostics and treatment for childhood tuberculosis, alongside reduced transmission, improved infection detection, and preventive therapy for exposed children, especially those under 5 years.

## 1. Introduction

Tuberculosis is a chronic infectious disease caused by *Mycobacterium tuberculosis* (MTB). It primarily targets the pulmonary system and remains a major global public health challenge, particularly among children [1]. The World Health Organisation (WHO) estimated that in 2022, approximately 1.25 million children (aged 0–14 years) were diagnosed with tuberculosis, accounting for 12% of the total global burden [2].

Evidence suggests that the true incidence of childhood tuberculosis substantially exceeds official notification data indicate. More than 60% of cases are estimated to be unreported or undiagnosed, nearly twice the proportion observed in adults [3]. This under-ascertainment is largely attributable to the non-specific clinical manifestations of tuberculosis in children, which frequently overlap with other common paediatric conditions, including pneumonia, malnutrition, and HIV-associated illnesses [4]. In addition, the emergence and spread of multidrug-resistant tuberculosis (MDR-TB) and extensively drug-resistant tuberculosis (XDR-TB) further complicate effective treatment and control [5].

Diagnosing tuberculosis in children remains difficult because symptoms are often non-specific and overlap with common paediatric illnesses [6]. The disease is typically paucibacillary, which limits microbiological confirmation and reduces the yield of smear microscopy and molecular assays when specimens are suboptimal [7]. Many young children cannot expectorate sputum, and diagnosis often depends on alternative specimens such as gastric aspirates or induced sputum, which require trained staff and may be difficult to implement consistently in routine settings [8]. Stool-based testing has expanded access to bacteriological confirmation on the GeneXpert platform and is supported by WHO guidance for Xpert MTB/RIF and Xpert MTB/RIF Ultra in children, but uptake remains uneven in high-burden settings [9]. Radiographic features may be subtle and interpretation varies across readers, while tests for latent infection do not reliably distinguish latent infection from active disease [10]. These constraints contribute to under-notification and delayed treatment, reinforcing the need for comparable burden estimates to inform paediatric tuberculosis control strategies worldwide.

Most epidemiological research on tuberculosis has focused on adults, whereas paediatric studies are often limited to specific regions or time periods. This study provides a comprehensive global, regional, and national assessment of childhood tuberculosis using data from the Global Burden of Disease (GBD) 2021 study [11].

## 2. Methods

### 2.1. Case Definition and Data Collection

Data on tuberculosis were obtained from the GBD 2021 study using the Global Health Data Exchange query tool (http://ghdx.healthdata.org/gbd-results-tool, accessed on 12 February 2025). Detailed descriptions of the GBD methods for estimating mortality and burden have been published elsewhere [11,12]. Consistent with the WHO definition and most national tuberculosis reports, children were defined as individuals aged 0–14 years [13]. The tuberculosis case definition includes all forms of tuberculosis (pulmonary tuberculosis and extrapulmonary tuberculosis), either bacteriologically confirmed or clinically diagnosed [11].

The classification of tuberculosis was based on the International Classification of Diseases (ICD) coding system. The relevant ICD-10 codes include A10-A19.9, B90-B90.9, K67.3, K93.0, M49.0, and P37.0; corresponding ICD-9 codes are 010-019.9, 137-137.9, 138.0, 138.9, 139.9, 320.4, and 730.4-730.6 [11]. The GBD 2021 separately estimated the incidence and disability-adjusted life years (DALYs) of MDR-TB and XDR-TB. Definition of MDR-TB without extensive drug resistance and XDR-TB are provided in the supplement [11].

To quantify the burden of tuberculosis, GBD 2021 synthesised multiple data sources, including annual case notifications, prevalence survey data, and estimated cause-specific mortality rates among individuals living with HIV and those not living with HIV [14]. For MDR-TB and XDR-TB, data inputs included: (1) counts of TB cases; (2) new and previously treated TB cases with drug susceptibility testing (DST) results for isoniazid and rifampicin; (3) MDR-TB cases with DST results for second-line drugs from routine surveillance systems and surveys reported to the WHO; and (4) evidence on the association between HIV and MDR-TB risk derived from the literature. These inputs were integrated using Bayesian meta-regression tools (DisMod-MR 2.1) and spatiotemporal Gaussian process regression to generate estimates for paediatric populations, accounting for regional patterns and data availability. HIV infection status is a key covariate within the GBD modelling framework for tuberculosis. The estimation models for tuberculosis incidence and mortality incorporate country-level HIV prevalence as a predictive covariate to adjust the burden estimates accordingly. However, the final estimates presented in the GBD results tool and used in this analysis represent the aggregate burden for all children aged 0–14 years, as the publicly available data do not provide results stratified by HIV status. GBD 2021 conducted a systematic literature review; the detailed search strategy is provided in the supplement.

The sociodemographic index (SDI) is a composite measure defined as the geometric mean of three rescaled indicators (ranging from 0 to 1): total fertility rate under age 25, mean education for individuals aged ≥15 years, and country’s lag-distributed income per capita [11]. The GBD 2021 study categorised 204 countries into five SDI levels: low (<0.46), low-middle (0.46 ≤ SDI < 0.61), middle ((0.61 ≤ SDI < 0.71), high-middle (0.71 ≤ SDI < 0.81), and high (≥0.81) [11,15].

### 2.2. Statistical Analysis

The GBD 2021 study used the following formula to calculate the age-standardised rates (ASR) per 100,000 population:

$$ASR=\frac{\sum_{i=1}^{N}\alpha_{i}w_{i}}{\sum_{i=1}^{N}w_{i}}$$

where $\alpha_{i}$ and $w_{i}$ refer to the age-specific rate and number of individuals (or weight) in the i age group within the chosen GBD standard population. *N* represents the number of age groups. Temporal trends in ASR and DALY rate were quantified using the estimated annual percentage change (EAPC), which is widely applied in GBD trend analyses because it accounts for year-to-year variability and provides a summary measure of change over time [16,17]. EAPC and its 95% confidence interval (CI) were derived from a log-linear regression model:

y=α+βx+ε

$$EAPC=100\times (e^{\beta }-1)$$

where y = ln (ASR), and x = calendar year.

An increasing trend was defined when the EAPC and its 95% CI were both >0, and a decreasing trend when both were <0. If the 95% CI included 0, the trend was considered not statistically significant. GBD 2021 produces model-based estimates for ASIR and DALY using Bayesian and ensemble modelling frameworks. Uncertainty for these quantities is reported as 95% uncertainty intervals (UIs), defined as the 2.5th and 97.5th percentiles of 1000 posterior draws that jointly reflect sampling variability and model uncertainty. In contrast, 95% confidence intervals (CIs) in this study refer to frequentist uncertainty around parameters estimated in regression models and are not interchangeable with GBD UI.

Smoothing spline models were used to assess the relationship between childhood tuberculosis burden and SDI across 21 regions. The expected value line was defined as the spline-predicted mean ASR at each SDI level, estimated by fitting the model to pooled region–year observations (1990–2021) and then evaluating the fitted curve across the observed SDI range. These lines represent the expected disease burden for a given SDI level based on global trends. Regions or SDI groups positioned above the line indicate a burden higher than expected for their level of development, while those below the line indicate a lower-than-expected burden. Robust fitting was used to reduce the influence of outlier by iterative reweighting of residuals. Predictions were restricted to the observed SDI range to minimise extrapolation bias. Spearman correlation analyses were conducted to estimate correlation coefficients (*r*) and *p* values for association between ASR and SDI, with statistical significance defined as *p* < 0.05. All statistical analyses were conducted using R 4.2.1 (R Foundation for Statistical Computing, Vienna, Austria).

## 3. Results

### 3.1. Global Level

In 2021, an estimated 759,300 new cases of tuberculosis among children aged 0–14 years were reported globally, corresponding to an age-standardised incidence rate (ASIR) of 37.7 per 100,000 population. From 1990 to 2021, the global ASIR of tuberculosis exhibited a declining trend, with an EAPC of −2.61% (95% CI: −2.74 to −2.47) (Table 1). During the same period, tuberculosis-related DALY totalled 6,646,694, with an age-standardised DALY rate of 330.4 per 100,000 population. The age-standardised DALY rate also declined consistently between 1990 and 2021 (EAPC = −4.38; 95% CI: −4.61 to −4.14) (Table S1).

### 3.2. Regional Level

Regionally, the highest absolute number of incident cases in 2021 was concentrated in South Asia (194,113), followed by Eastern Sub-Saharan Africa (136,955) and Western Sub-Saharan Africa (131,997) (Table 1). Among all GBD regions, Southern Sub-Saharan Africa consistently had the highest burden of tuberculosis incidence, with an ASIR of 164.3 per 100,000 population in 2021, followed by Central Sub-Saharan Africa and Eastern Sub-Saharan Africa (Table 1).

From 1990 to 2021, an increasing trend in ASIR of tuberculosis was observed only in High-income North America (EAPC = 1.12, 95% CI: 0.61 to 1.64). In 2021, the highest age-standardised DALY rates (per 100,000) were recorded in Central Sub-Saharan Africa (1425.2), Southern Sub-Saharan Africa (1051.2), and Eastern Sub-Saharan Africa (850.0). Over the same period, all GBD regions exhibited a significant decreasing trend in age-standardised DALY rate for tuberculosis.

### 3.3. National Level

**Figure 1:**
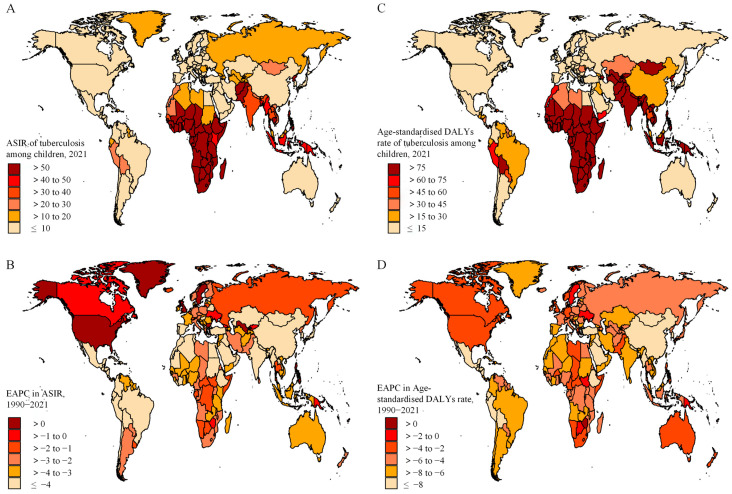
(**A**) Age-standardised incidence rates (ASIR) of tuberculosis among children per 100,000 population in 2021; (**B**) estimated annual percentage change in ASIR from 1990 to 2021; (**C**) age-standardised DALY rate of tuberculosis among children per 100,000 population in 2021; (**D**) estimated annual percentage change in age-standardised DALY rate from 1990 to 2021. DALY: disability-adjusted life years.

In 2021, the national ASIR of tuberculosis ranged from 0.5 to 270.7 cases per 100,000 population. Lesotho (270.7), Central African Republic (264.5), and Kingdom of Eswatini (188.6) reported the highest ASIR of tuberculosis (Figure 1). The highest age-standardised DALY rates were seen in the Central African Republic (5279.3), South Sudan (2742.9), and Somalia (2536.2). From 1990 to 2021, the largest increase in ASIR of tuberculosis was observed in the Philippines (EAPC = 4.44, 95% CI: 3.56 to 5.32), while the largest increase in age-standardised DALY rate was found in Zimbabwe (0.85, 0.52 to 1.17).

### 3.4. Age and Sex Patterns

**Figure 2:**
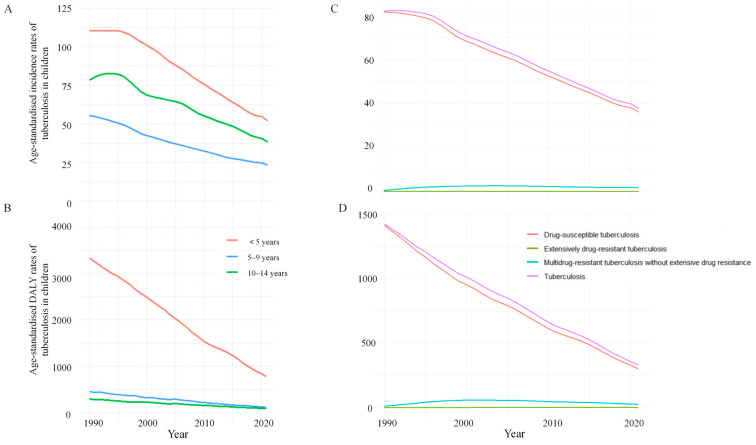
Age-standardised incidence rates (**A**) and age-standardised DALY rate (**B**) of tuberculosis among children in different age groups from 1990 to 2021. Age-standardised incidence rates (**C**) and age-standardised DALY rate (**D**) of different types of tuberculosis among children from 1990 to 2021.

From 1990 to 2021, both ASIR and age-standardised DALY rate decreased across all paediatric age groups (Table 1 and Table S1). In 2021, children aged <5 years accounted for the highest numbers of incident cases and DALYs, and they also exhibited the highest ASIR and age-standardised DALY rate. The largest decrease in ASIR occurred among children aged 5–9 years (EAPC = −2.82, 95% CI: −2.86 to −2.77), while the largest decrease in age-standardised DALY rate was observed in children less than 5 years of age (−4.47, −4.72 to −4.22). Declining trends in both ASIR and age-standardised DALY rate were observed for males and females over the study period (Figure 2).

### 3.5. Association with the Sociodemographic Index (SDI)

In 2021, the low-SDI region had the highest ASIR (70.3, 95% UI: 55.4–87.8) and age-standardised DALY rate (878.3, 95% UI: 635.7–1146.1) of tuberculosis. From 1990 to 2021, both ASIR and the age-standardised DALY rate decreased significantly across all SDI regions. The largest reduction in ASIR occurred in the high-middle SDI region (EAPC = −4.58, 95% CI: −4.69 to −4.48), whereas the largest reduction in the age-standardised DALY rate occurred in the high SDI region (EAPC = −8.23, 95% CI: −8.53 to −7.92).

**Figure 3:**
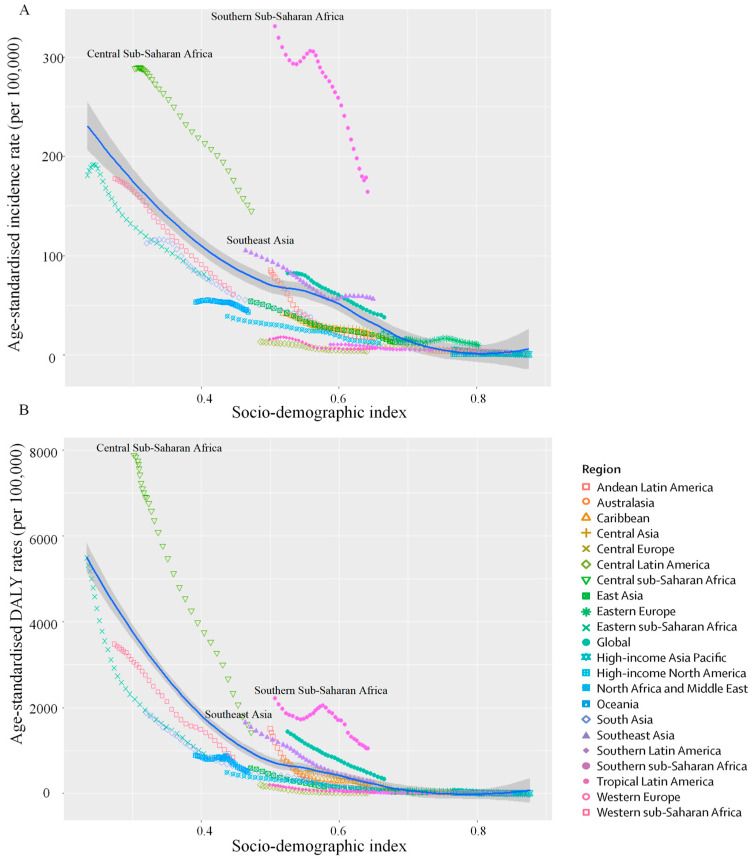
(**A**) Age-standardised incidence rates of tuberculosis among children for the 21 GBD regions by sociodemographic index, 1990–2021; (**B**) Age-standardised DALY rate of tuberculosis among children for the 21 GBD regions by sociodemographic index, 1990–2021. The solid line indicates the spline-based expected age-standardised rate as a function of SDI (model-predicted mean), estimated from all region–year observations. Regions above the solid line represent a higher-than-expected burden, and regions below the line show a lower-than-expected burden. DALYs: disability-adjusted life years; GBD: Global Burden of Diseases.

At the regional level, ASIR and age-standardised DALY rate of tuberculosis among children decreased exponentially with increasing SDI (Figure 3A,B). Central Sub-Saharan Africa, Southern Sub-Saharan Africa, and Southeast Asia exhibited higher-than-expected ASIR and age-standardised DALY rate based on their SDI from 1990 to 2021. In contrast, regions below the expected line may reflect comparatively better tuberculosis control performance at similar SDI levels. Negative correlations were found between ASIR, age-standardised DALY rate, and SDI among regions (*ρ* = −0.844, *p* < 0.001; *ρ* = −0.893, *p* < 0.001).

### 3.6. TB Drug Resistance Pattern

**Figure 4:**
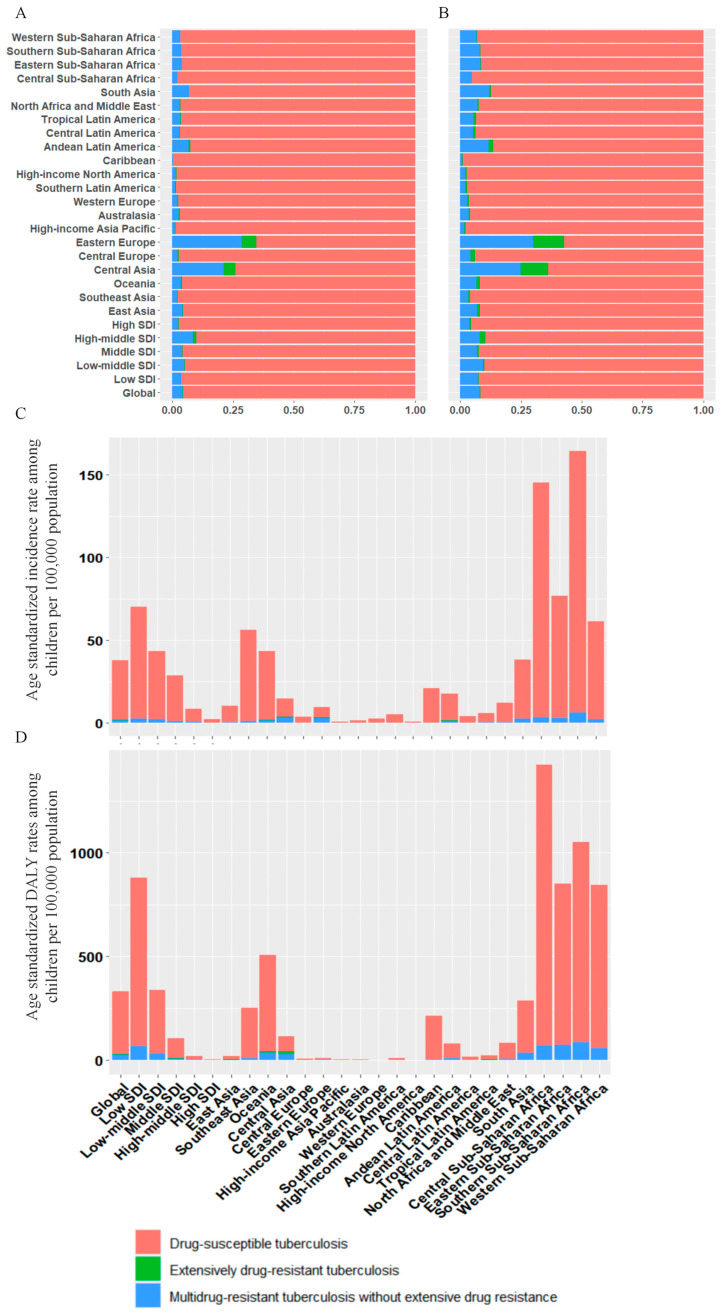
Age-standardised incidence and DALY rate of tuberculosis and proportions of incident cases and DALYs contributed by tuberculosis among children, globally and for 21 GBD regions, 2021. (**A**) Proportions of incident cases globally and for 21 GBD regions; (**B**) Proportions of DALYs globally and for 21 GBD regions; (**C**) Age-standardised incidence rate globally and for 21 GBD regions; (**D**) Age-standardised DALY rate globally and for 21 GBD regions.

The ASIR of different tuberculosis resistance profiles varied substantially across regions in 2021 (Figure 4). Globally, an estimated 725,593 incident cases of drug-susceptible tuberculosis were reported in 2021, corresponding to an ASIR of 36.1 per 100,000 population (Table 1). From 1990 to 2021, the ASIR for drug-susceptible tuberculosis declined globally (EAPC = −2.70, 95% CI −2.82 to −2.59), with High-income North America being the only region showing an increase trend (EAPC = 1.15, 95% CI: 0.64 to 1.66).

Globally, an estimated 32,515 incident MDR-TB cases were reported in 2021, resulting in an ASIR of 1.6 per 100,000 population (Table 1). From 1990 to 2021, there was no significant change in ASIR of MDR-TB (EAPC = 1.18, 95% CI: −0.16 to 2.54). However, 8 of the 21 GBD regions showed an increasing trend in ASIR of MDR-TB, with the largest increasing trend observed in Oceania (11.99,10.49 to 13.52), followed by Central Asia, South Asia, and Eastern Europe. In addition, significant increases were observed in low (2.77, 0.97 to 4.61) and low-middle SDI (4.33, 2.04 to 6.67) regions. In contrast, MDR-TB ASIR declined significantly in middle, high-middle, and high SDI regions.

## 4. Discussion

From 1990 to 2021, the ASIR and age-standardised DALY rate of tuberculosis among children declined in nearly all regions. However, eight GBD regions experienced significant increases in the ASIR of MDR-TB, with Oceania reporting the largest rise, followed by Central Asia, South Asia, and Eastern Europe. The burden of tuberculosis among children exhibited pronounced geographical heterogeneity, characterised by a strong negative correlation between tuberculosis burden and the SDI.

Sub-Saharan Africa remains the region with the highest tuberculosis burden among children, consistent with WHO estimates. The WHO reports that 17 of the 30 countries with the highest global tuberculosis burden are in Africa [18]. According to the latest estimates in 2022, Africa accounts for approximately one-third of tuberculosis cases among children aged 0–15 years worldwide (about 320,000 cases). This disproportionate burden is plausibly driven by a convergence of factors, including high HIV prevalence, fragile healthcare systems, poverty, malnutrition, and overcrowded living conditions [19,20]. A systematic review and meta-analysis estimated that about 32% of tuberculosis patients in sub-Saharan Africa are HIV-positive, indicating a high tuberculosis/HIV co-infection burden among tuberculosis patients [21]. The high prevalence of tuberculosis/HIV co-infection among adults increase the risk of households and community transmission, placing children at particular risk. Additionally, malnutrition not only heightens the likelihood of progression to active tuberculosis following MTB infection but also exacerbates disease severity and increases mortality risk. Globally, malnutrition is estimated to contribute to up to 19% of tuberculosis cases [18].

This study reveals that, despite the relatively low incidence of tuberculosis among children in High-income North America, there has been a noticeable upward trend from 1990 to 2021. This pattern is largely attributed to the impact of immigration and imported tuberculosis, as reported in multiple studies. Pang et al. [22] demonstrated that tuberculosis incidence among foreign-born children was 32 times higher than among US-born children with US-born parents, while US-born children with foreign-born parents had rates six times higher. Similarly, data from Canadian hospitals indicate that approximately 60% of non-Indigenous children diagnosed with tuberculosis were born outside Canada, with the majority having at least one foreign-born parent [23]. Children migrating from high-burden regions may carry latent Mycobacterium tuberculosis infections (LTBI), which can progress to active tub disease more readily in children because of immune immaturity [24]. In addition, the increased use of advanced diagnostic tools, such as Xpert MTB/RIF, has likely improved case detection and reporting in recent years, contributing to the observed increase [25]. Furthermore, children born in high-income countries who travel to visit relatives in high tuberculosis burden countries of origin represent another key at-risk group often overlooked in surveillance [26]. These trips can involve prolonged stays in settings with high community transmission, significantly increasing the risk of Mycobacterium tuberculosis infection. Upon return, these children may develop active TB disease, contributing to the locally acquired case count [27]. This underscores the importance of paediatricians and public health systems in low-incidence countries incorporating detailed travel history into risk assessments and considering pre- or post-travel screening and targeted provision of preventive therapy for eligible child travellers.

Similar to previous evidence, this study found the burden of tuberculosis is highest among children under 5 years of age [28]. Once infected with MTB, young children are more likely than adults to develop active tuberculosis and to progress rapidly [28]. Infants and young children are more likely to develop severe forms of tuberculosis, such as tuberculous meningitis, and face challenges in diagnosis due to the paucibacillary nature of the disease and difficulties in obtaining sputum samples [29]. The observed disparity in age-specific trends, where 5–9-year-olds exhibit lower tuberculosis incidence but higher DALY rate compared to 10–14-year-olds, likely reflects differences in disease progression and healthcare access [30]. Younger children may experience more rapid progression to severe extrapulmonary forms due to immature immune systems, leading to disproportionate disability despite fewer cases [31,32]. This is consistent with clinical evidence that childhood tuberculosis in those under age 10 often presents as disseminated disease and is associated with higher mortality and neurological complications [33,34,35]. In contrast, the higher incidence in 10–14-year-olds may be partly explained by increased exposure in school settings, while more adult-like pulmonary presentations and relatively mature immune responses may facilitate earlier diagnosis and more effective treatment, thereby reducing disability per case [36].

Reducing transmission to the paediatric population is essential to accelerate declines in childhood tuberculosis burden [37]. Because most paediatric infections arise from recent exposure in households or other close-contact settings, systematic contact investigation and timely identification of tuberculosis infection among exposed children should be prioritised, particularly for those under 5 years of age who have a higher risk of rapid progression and severe disease [38]. Prompt initiation of tuberculosis preventive treatment for eligible child contacts, coupled with linkage to care and follow-up to support completion, can substantially reduce incident disease and tuberculosis-related mortality [39]. Integrating contact management, infection testing where feasible, and preventive therapy delivery into routine maternal and child health services in high-burden settings may improve coverage and equity while strengthening overall tuberculosis control.

The rising incidence of MDR-TB among children in regions such as Oceania, South Asia, and Central Asia likely reflects a combination of geographic constraints, socioeconomic conditions, and health-system challenges. In Oceania, remote islands settings can face substantial barriers to MDR-TB diagnosis and treatment, including limited laboratory capacity and unstable drug supplies [40]. In 2015, nearly 1% of the population on Daru Island, Papua New Guinea, was diagnosed annually with MDR-TB, likely underestimating the true burden due to the absence of active case-finding [41]. In South Asia, a large treatment gap persists, with at least 70% of MDR-TB cases remaining untreated [42]. Rapid urbanisation, high population density, rising diabetes rates, unregulated private healthcare, and escalating drug resistance contribute to the growing epidemic [43]. Additionally, smoking, occupational lung diseases, and air pollution further increase tuberculosis vulnerability [42]. In Central Asia, countries like Kazakhstan and Uzbekistan have a high MDR-TB burden linked to the legacy of the Soviet-era healthcare system [44]. Estimates suggest that 13–26% of new adult tuberculosis cases are MDR-TB, and similar patterns may occur among children [45]. Treatment interruptions, inadequate protocols, and poor infection control in settings like prisons exacerbate the spread [45].

The observed increases in paediatric MDR-TB incidence in several regions likely reflect a combination of ongoing transmission and programmatic weaknesses in detection and treatment. In many high-burden settings, incomplete access to timely drug susceptibility testing and delayed initiation of effective regimens can prolong infectiousness among source cases and facilitate household and community transmission to children [46]. Treatment interruptions, inconsistent drug supply chains, suboptimal adherence support, and care fragmentation may further amplify the emergence and spread of resistance [47]. From a health-system perspective, rising paediatric MDR-TB increases demand for rapid molecular diagnostics and DST capacity, specialist clinical management, longer and more expensive treatment courses, child-appropriate formulations, monitoring for adverse events, and robust procurement and pharmacovigilance systems [48]. These pressures can widen inequities in access to diagnosis and treatment and may displace resources from routine TB prevention and case-finding. Future control strategies should prioritise earlier detection and resistance profiling through expanded access to rapid molecular testing and DST, strengthened contact investigation and preventive therapy for child contacts, improved linkage to care and treatment completion, and infection prevention and control in high-risk congregate settings. The COVID-19 pandemic may also have contributed to changes in tuberculosis notifications and apparent trends through reduced care-seeking, diagnostic delays, and service disruptions, particularly in 2020–2021 [49]. Therefore, late-period estimates should be interpreted in light of potential pandemic-related under-detection and reporting artifacts.

The negative correlation between tuberculosis burden among children and SDI aligns with existing studies, suggesting that lower socioeconomic development is a key driver of tuberculosis burden [50,51]. Regions with lower SDI often face inadequate healthcare infrastructure, limited access to quality diagnostics, and insufficient treatment coverage, which contribute to the persistence and spread of tuberculosis, particularly among children [50]. In low- and middle-income countries (LMICs), malnutrition and high HIV prevalence significantly exacerbate the burden of tuberculosis. These factors not only increase the risk of contracting tuberculosis but also worsen disease progression and treatment outcomes [51]. The significant increase in MDR-TB incidence in low- and low-middle SDI regions is consistent with findings from other studies, which highlight how poverty, overcrowding, malnutrition, and weak healthcare systems exacerbate drug resistance. These findings underscore the urgent need for targeted interventions, including improved healthcare access, strengthened tuberculosis control programs, and socioeconomic development, to address the disproportionate burden of tuberculosis and MDR-TB among children in low-resource settings.

This study has several limitations. First, GBD 2021 estimates depend on the availability and quality of primary data, and paediatric tuberculosis is especially vulnerable to under-diagnosis and under-reporting because bacteriological confirmation is often not feasible and surveillance systems are incomplete in many high-burden settings. These deficiencies may lead to systematic underestimation of incidence and DALYs, particularly in low- and low-middle SDI countries. Second, estimates of paediatric MDR-TB and XDR-TB are limited by incomplete and heterogeneous drug susceptibility testing coverage and sparse child-specific DST data. Because childhood tuberculosis is frequently paucibacillary and often diagnosed clinically without bacteriological confirmation, resistance patterns in children are commonly inferred through modelling that borrows strength across locations and years and, in part, draws on adult surveillance data and covariates. These features increase uncertainty, may reduce accuracy for specific settings, and may not fully capture rapid epidemiologic changes. Third, substantial inter-country heterogeneity exists in diagnostic algorithms, access to imaging and rapid molecular testing, case notification practices, and the completeness of vital registration and cause-of-death attribution. Such heterogeneity can introduce measurement differences that affect cross-country comparisons and trend interpretation. Fourth, the COVID-19 pandemic likely disrupted tuberculosis services and reporting in parts of the study period, potentially affecting notification-based inputs and leading to transient under-detection. Finally, the GBD cause-level framework focuses on active tuberculosis disease and does not fully capture the burden of latent tuberculosis infection, which is an important driver of future paediatric morbidity. Accordingly, the estimated trends should be interpreted with appropriate caution.

Targeted interventions remain essential, particularly in high-burden areas such as Sub-Saharan Africa, South Asia, and Oceania. Achieving elimination targets will require strengthening childhood tuberculosis prevention, diagnosis, and treatment by expanding diagnostic capacity, improving access to effective regimens, and addressing rising MDR-TB and XDR-TB burdens, while also reducing transmission through systematic contact investigation and improved detection and preventive treatment of infection among paediatric contacts, especially those under 5 years. Strengthening surveillance systems is vital to monitor trends and sustain reductions in morbidity and mortality.

## Figures and Tables

**Table 1:** Childhood tuberculosis incidence in 1990 and 2021 and temporal trends, 1990–2021.

	Incidence				
	Cases (*n*), 1990	ASR per 100,000, 1990	Cases (*n*), 2021	ASR per 100,000, 2021	EAPC 1990–2021
**Overall**	1,428,396 (1,115,872 to 1,788,301)	82.1 (64.2 to 102.8)	759,300 (596,055 to 949,256)	37.7 (29.6 to 47.2)	−2.61 (−2.74 to −2.47)
**Sex**					
Male	273,361 (448,643 to 720,236)	64.2 (50.2 to 80.6)	316,171 (247,282 to 398,948)	30.5 (23.8 to 38.4)	−2.47 (−2.57 to −2.36)
Female	855,034 (664,980 to 1,068,748)	101.1 (78.6 to 126.4)	443,130 (349,074 to 549,631)	45.5 (35.9 to 56.5)	−2.70 (−2.86 to −2.53)
**Age group**					
<5	684,495 (528,175 to 867,475)	110.4 (85.2 to 139.9)	342,720 (266,651 to 432,590)	52.1 (40.5 to 65.7)	−2.60 (−2.79 to −2.42)
5–9	323,164 (207,543 to 485,446)	55.4 (35.6 to 83.2)	161,788 (103,812 to 244,585)	23.5 (15.1 to 35.6)	−2.82 (−2.86 to −2.77)
10–14	420,736 (263,400 to 604,685)	78.5 (49.2 to 112.9)	254,793 (158,010 to 374,718)	38.2 (23.7 to 56.2)	−2.47 (−2.63 to −2.31)
**TB drug resistance pattern**					
Drug-susceptible tuberculosis	1,420,310 (1,107,523 to 1,777,803)	81.7 (63.7 to 102.2)	725,593 (568,127 to 910,846)	36.1 (28.2 to 45.3)	−2.70 (−2.82 to −2.59)
Multidrug-resistant tuberculosis without extensive drug resistance	8086 (3542 to 19,038)	0.5 (0.2 to 1.1)	32,515 (20,968 to 51,288)	1.6 (1.0 to 2.6)	1.18 (−0.16 to 2.54)
Extensively drug-resistant tuberculosis	NA	NA	1193 (806 to 1813)	0.1 (0.0 to 0.1)	NA
**SDI level**					
Low SDI	381,303 (305,165 to 468,461)	166.6 (133.3 to 204.6)	323,363 (255,117 to 404,199)	70.3 (55.4 to 87.8)	−2.93 (−3.11 to −2.75)
Low-middle SDI	551,050 (412,873 to 700,861)	116.7 (87.5 to 148.5)	250,778 (194,694 to 317,548)	43.2 (33.6 to 54.8)	−3.42 (−3.57 to −3.27)
Middle SDI	390,645 (303,096 to 491,120)	67.7 (52.5 to 85.1)	161,888 (125,273 to 205,207)	28.6 (22.1 to 36.2)	−2.87 (−2.94 to −2.81)
High-middle SDI	92,430 (71,353 to 117,196)	33.8 (26.1 to 42.8)	19,079 (14,353 to 24,843)	8.3 (6.2 to 10.8)	−4.58 (−4.69 to −4.48)
High SDI	12,248 (9559 to 15,445)	6.6 (5.1 to 8.3)	3725 (2767 to 4915)	2.2 (1.6 to 2.8)	−3.72 (−3.9 to −3.54)
**Region**					
East Asia	178,503 (140,906 to 221,858)	54.1 (42.7 to 67.3)	27,575 (21,333 to 34,933)	10.3 (8.0 to 13.1)	−5.30 (−5.47 to −5.13)
Southeast Asia	179,068 (138,347 to 227,011)	104.9 (81.0 to 133.0)	96,962 (75,163 to 122,721)	56.2 (43.5 to 71.1)	−2.18 (−2.44 to −1.91)
Oceania	1431 (1069 to 1859)	53.4 (39.9 to 69.4)	2201 (1650 to 2852)	43.3 (32.5 to 56.1)	−0.6 (−0.73 to −0.46)
Central Asia	8286 (6337 to 10,554)	33.2 (25.4 to 42.2)	4087 (3216 to 5226)	14.8 (11.6 to 18.9)	−2.11 (−2.46 to −1.75)
Central Europe	3252 (2442 to 4246)	11.0 (8.3 to 14.4)	678 (515 to 893)	3.8 (2.9 to 5.0)	−3.4 (−3.48 to −3.33)
Eastern Europe	12,179 (8991 to 16,333)	23.7 (17.5 to 31.7)	3430 (2405 to 4841)	9.7 (6.8 to 13.7)	−1.52 (−1.97 to −1.07)
High-income Asia Pacific	1774 (1456 to 2201)	5 (4.1 to 6.3)	191 (136 to 263)	0.9 (0.6 to 1.2)	−5.62 (−5.8 to −5.43)
Australasia	160 (118 to 215)	3.5 (2.6 to 4.7)	90 (68 to 118)	1.6 (1.2 to 2.1)	−2.99 (−3.15 to −2.83)
Western Europe	3971 (2932 to 5351)	5.6 (4.1 to 7.5)	1741 (1248 to 2417)	2.6 (1.8 to 3.5)	−2.14 (−2.36 to −1.93)
Southern Latin America	1617 (1210 to 2093)	10.8 (8.1 to 14)	740 (544 to 998)	5.1 (3.8 to 6.9)	−2.82 (−3.02 to −2.61)
High-income North America	487 (351 to 654)	0.8 (0.6 to 1.1)	518 (384 to 683)	0.8 (0.6 to 1.0)	1.12 (0.61 to 1.64)
Caribbean	4762 (3745 to 5938)	41.7 (32.8 to 52)	2402 (1843 to 3038)	20.9 (16.0 to 26.4)	−2.26 (−2.42 to −2.1)
Andean Latin America	12,661 (9578 to 16,226)	85.2 (64.5 to 109.3)	3165 (2396 to 4159)	17.5 (13.2 to 23)	−5.62 (−5.95 to −5.28)
Central Latin America	8684 (6680 to 10,858)	13.5 (10.4 to 16.9)	2666 (1998 to 3422)	4.2 (3.1 to 5.4)	−4.38 (−4.68 to −4.09)
Tropical Latin America	8300 (6327 to 10,700)	15.5 (11.8 to 20)	3018 (2230 to 3913)	6.0 (4.4 to 7.8)	−4.52 (−5.25 to −3.79)
North Africa and Middle East	54,427 (42,582 to 67,754)	38.7 (30.3 to 48.2)	22,027 (16,895 to 28,087)	12.0 (9.2 to 15.3)	−3.6 (−3.89 to −3.31)
South Asia	487,209 (353,180 to 642,547)	112.4 (81.5 to 148.3)	194,113 (147,300 to 254,329)	38.3 (29.1 to 50.2)	−3.91 (−4.08 to −3.73)
Central Sub-Saharan Africa	73,028 (57,964 to 90,658)	288.7 (229.1 to 358.4)	85,205 (66,777 to 105,554)	145.2 (113.8 to 179.9)	−2.18 (−2.48 to −1.88)
Eastern Sub-Saharan Africa	163,904 (132,289 to 199,174)	181 (146.1 to 219.9)	136,955 (106,318 to 174,344)	76.8 (59.6 to 97.7)	−3.07 (−3.25 to −2.88)
Southern Sub-Saharan Africa	68,464 (54,971 to 84,660)	330.9 (265.7 to 409.2)	39,541 (29,922 to 50,817)	164.3 (124.3 to 211.2)	−1.99 (−2.31 to −1.67)
Western Sub-Saharan Africa	156,227 (125,487 to 191,979)	177.8 (142.8 to 218.5)	131,997 (105,932 to 163,521)	61.5 (49.3 to 76.1)	−3.41 (−3.7 to −3.11)

ASR, age-standardised rate; EAPC, estimated annual percentage change. Incidence data for extensively drug-resistant tuberculosis in 1990 were not available in the GBD 2021 database; therefore, the EAPC could not be calculated. The corresponding entries are marked as “NA” (not available).

## Data Availability

The data used for the analyses in the study are publicly available at https://ghdx.healthdata.org/gbd-results-tool, accessed on 12 February 2025.

## Supplementary Materials

The following supporting information can be downloaded at https://www.mdpi.com/article/10.3390/tropicalmed11020043/s1, Table S1. The disability-adjusted life years (DALYs) of tuberculosis in children aged 0–14 years in 1990 and 2021 and estimated annual percentage change from 1990 to 2021 (generated from data available at https://ghdx.healthdata.org/gbd-results-tool) (accessed on 12 February 2025); Table S2. The incidence of drug-susceptible tuberculosis in children aged 0–14 years in 1990 and 2021 and estimated annual percentage change from 1990 to 2021 (generated from data available at https://ghdx.healthdata.org/gbd-results-tool) (accessed on 12 Feb 2025); Table S3. The disability-adjusted life years (DALYs) of drug-susceptible tuberculosis in children aged 0–14 years in 1990 and 2021 and estimated annual percentage change from 1990 to 2021 (generated from data available at https://ghdx.healthdata.org/gbd-results-tool) (accessed on 12 February 2025); Table S4. The incidence of multidrug-resistant tuberculosis in children aged 0–14 years in 1990 and 2021 and estimated annual percentage change from 1990 to 2021 (generated from data available at https://ghdx.healthdata.org/gbd-results-tool) (accessed on 12 February 2025); Table S5. The disability-adjusted life years (DALYs) of multidrug-resistant tuberculosis in children aged 0–14 years in 1990 and 2021 and estimated annual percentage change from 1990 to 2021 (generated from data available at https://ghdx.healthdata.org/gbd-results-tool) (accessed on 12 February 2025); Table S6. The change in age-standardised incidence rate of tuberculosis among children aged 0–14 years from 1990 to 2021 at national level. Table S7. The change in age-standardised DALY rate of tuberculosis among children aged 0–14 years from 1990 to 2021 at national level.

## Abbreviations

GBD: Global Burden of Disease; DALYs: disability-adjusted life years; EAPCs: estimated annual percentage changes; ASIR: age-standardised incidence rates; SDI: sociodemographic index; WHO: World Health Organisation; MDR-TB: multidrug-resistant tuberculosis; XDR-TB: extensively drug-resistant tuberculosis; ICD: International Classification of Diseases; LTBI: Mycobacterium tuberculosis infections; LMICs: low- and middle-income countries.
