# Supplementary material for: Global, Regional, and National Burden of Tuberculosis Among Children: A Population-Based Study
Source: Trop Med Infect Dis. 2026 Feb 5;11(2):43. doi: 10.3390/tropicalmed11020043 (PMC12944865; doi:10.3390/tropicalmed11020043)
Supplement: Supplementary file 1 [file tropicalmed-11-00043-s001.zip › tropicalmed-4026599-supplementary.pdf]

SUPPLEMENTARY MATERIAL

Supplement Methods:

Search strategies

Pubmed: ("tuberculosis"[MeSH] OR tuberculosis[Title/Abstract] OR TB[Title/Abstract] OR Mycobacterium tuberculosis[Title/Abstract] AND prevalence[Title/Abstract] AND ("2019/02/01"[PDAT] : "2020/04/07"[PDAT]) NOT (animals\{SESH] NOT humans[MESH])

The targeted literature review was restricted to publications from 2019-2020 because it was designed to identify the most recent evidence needed for the GBD 2021 update of drug-resistant tuberculosis parameters, particularly where routine surveillance and earlier syntheses had limited coverage. Evidence from earlier years had been incorporated through prior GBD iterations and ongoing WHO-reported surveillance and survey data; therefore, the 2019-2020 window was selected to capture incremental, contemporary data consistent with the GBD 2021 compilation timeline.

Definition

MDR-TB without extensive drug resistance is defined as tuberculosis that is resistant to the two most effective first-line anti- tuberculosis drugs, isoniazid and rifampicin, but is not resistant to any fluoroquinolone or second-line injectable drugs (amikacin, kanamycin, or capreomycin). XDR-TB is characterized by resistance to isoniazid and rifampicin, in addition to resistance to any fluoroquinolone and any second-line injectable drugs. In contrast, drug-susceptible tuberculosis refers to tuberculosis that remains fully susceptible to isoniazid and rifampicin.

Disease model

The estimation of tuberculosis incidence in this study involves a two-tiered approach based on data quality. For countries with high-quality health systems (4- or 5-star ratings), age- and sex-specific tuberculosis notifications (including new and relapse cases) are used, with adjustments for missing data and case types (smear-positive, smear-negative, and extrapulmonary tuberculosis) through imputation and regression modeling. For countries with lower-quality data, a meta-regression with Bayesian priors, regularisation, and trimming (MR-BRT) is employed to predict mortality-to-incidence (MI) ratios, using the Healthcare Access and Quality (HAQ) Index as a covariate and anchoring estimates with historical cohort data. These MI ratios, combined with cause-specific mortality estimates, are used to compute tuberculosis incidence. To ensure alignment with case notifications, a correction factor based on the proportion of bacteriologically confirmed cases in high-quality countries is applied to low-quality countries, reducing overestimation. For disability-adjusted life years (DALYs) estimation, the model integrates incidence, prevalence, and mortality data with disability weights for tuberculosis and its sequelae. DALYs are calculated as the sum of years of life lost (YLL) due to premature mortality and years lived with disability (YLD) from tuberculosis-related health loss.

Supplement Results:

Table S1. The disability adjusted life years (DALYs) of tuberculosis in children aged 0-14 years in 1990 and 2021 and estimated annual percentage change from 1990 to 2021. (Generated from data available at https://ghdx.healthdata.org/gbd-results-tool)

	DALYs				
	Cases (n), 1990	ASR per 100 000, 1990	Cases (n), 2021	ASR per 100 000, 2021	EAPC 1990-2021
Overall	24892261 (20916336	1431.3 (1202.7 to	6646694 (5198363 to	330.4 (258.4 to	-4.38 (-4.61 to -4.14)

	to 28794984)	1655.7)	8305381)	412.8)	
Sex					
Male	10516279 (7246299 to 12825798)	1177.0 (811.0 to 1435.5)	2950646 (2185253 to 3911125)	284.2 (210.5 to 376.7)	-4.23 (-4.47 to -3.99)
Female	14375981 (12030819 to 17455756)	1700.0 (1422.7 to 2064.2)	3696048 (3024894 to 4511444)	379.6 (310.7 to 463.3)	-4.48 (-4.71 to -4.25)
Age group					
< 5	20651545 (17218154 to 23995317)	3331.2 (2777.4 to 3870.6)	5170594 (3841629 to 6653047)	785.6 (583.7 to 1010.8)	-4.47 (-4.72 to -4.22)
5-9	2659729 (2205115 to 3121955)	455.8 (377.9 to 535)	846644 (704486 to 1002624)	123.2 (102.5 to 145.9)	-4.11 (-4.31 to -3.91)
10-14	1580986 (1354258 to 1834824)	295.1 (252.8 to 342.5)	629456 (552253 to 705088)	94.4 (82.8 to 105.8)	-3.58 (-3.78 to -3.38)
TB drug resistance pattern					
Drug-susceptible tuberculosis	24703150 (20751972 to 28631645)	1420.4 (1193.2 to 1646.3)	6093957 (4702774 to 7595268)	302.9 (233.8 to 377.5)	-4.59 (-4.80 to -4.39)
Multidrug-resistant tuberculosis without extensive drug resistance	189110 (69056 to 436990)	10.9 (4.0 to 25.1)	530364 (235225 to 1002206)	26.4 (11.7 to 49.8)	0.51 (-1.05 to 2.09)
Extensively drug-resistant tuberculosis	NA	NA	22373 (9941 to 44342)	1.1 (0.5 to 2.2)	NA
SDI level					
Low SDI	10123077 (8189543 to 12008699)	4422.2 (3577.6 to 5246)	4042306 (2925842 to 5274590)	878.3 (635.7 to 1146.1)	-5.01 (-5.25 to -4.76)
Low-middle SDI	9529877 (7796723 to 11323145)	2018.6 (1651.5 to 2398.4)	1957056 (1610770 to 2327188)	337.5 (277.8 to 401.3)	-5.49 (-5.65 to -5.33)

Middle SDI	4522748 (3993677 to 4986302)	783.5 (691.9 to 863.9)	598722 (516868 to 694162)	105.6 (91.2 to 122.5)	-5.99 (-6.12 to -5.85)
High-middle SDI	648658 (553617 to 737369)	237.1 (202.3 to 269.5)	40826 (34410 to 47322)	17.7 (14.9 to 20.5)	-8.01 (-8.21 to -7.8)
High SDI	54949 (46929 to 64494)	29.6 (25.3 to 34.7)	3690 (2980 to 4556)	2.1 (1.7 to 2.6)	-8.23 (-8.53 to -7.92)
Region					
East Asia	1960782 (1666102 to 2300935)	594.5 (505.1 to 697.6)	47858 (39637 to 58118)	17.9 (14.8 to 21.7)	-10.59 (-10.74 to -10.43)
Southeast Asia	2808941 (2142026 to 3307656)	1645.1 (1254.5 to 1937.2)	433107 (362333 to 510531)	250.9 (209.9 to 295.7)	-5.89 (-6.02 to -5.76)
Oceania	23886 (15298 to 37336)	891.3 (570.8 to 1393.2)	25755 (15869 to 37301)	506.9 (312.3 to 734.2)	-1.50 (-1.88 to -1.13)
Central Asia	135138 (122122 to 149896)	540.7 (488.7 to 599.8)	31855 (25719 to 39318)	115.1 (92.9 to 142.1)	-4.92 (-5.33 to -4.5)
Central Europe	14977 (13908 to 16085)	50.8 (47.2 to 54.6)	1358 (1144 to 1582)	7.7 (6.5 to 8.9)	-6.31 (-6.67 to -5.96)
Eastern Europe	31040 (28658 to 33584)	60.3 (55.7 to 65.3)	3716 (3254 to 4225)	10.5 (9.2 to 11.9)	-4.53 (-5.67 to -3.37)
High-income Asia Pacific	16305 (13427 to 19685)	46.3 (38.1 to 55.9)	410 (352 to 479)	1.8 (1.6 to 2.1)	-10.05 (-10.44 to -9.66)
Australasia	218 (174 to 280)	4.8 (3.8 to 6.1)	81 (56 to 115)	1.4 (1.0 to 2.0)	-3.79 (-3.93 to -3.65)
Western Europe	3883 (3417 to 4454)	5.5 (4.8 to 6.3)	857 (631 to 1146)	1.3 (0.9 to 1.7)	-4.32 (-4.42 to -4.23)
Southern Latin America	13938 (13168 to 14807)	93.4 (88.2 to 99.2)	1474 (1267 to 1704)	10.2 (8.7 to 11.8)	-6.65 (-7.01 to -6.28)
High-income North America	2495 (2338 to 2686)	4.0 (3.8 to 4.4)	729 (571 to 935)	1.1 (0.9 to 1.4)	-3.38 (-3.96 to -2.80)

Caribbean	91514 (73860 to 118064)	801.9 (647.2 to 1034.5)	24460 (16912 to 39570)	212.6 (147.0 to 343.9)	-3.77 (-4.19 to -3.35)
Andean Latin America	223653 (182570 to 262860)	1505.9 (1229.3 to 1769.9)	14305 (11588 to 17617)	79.1 (64.0 to 97.4)	-9.36 (-9.68 to -9.04)
Central Latin America	126563 (118685 to 135470)	196.6 (184.3 to 210.4)	10382 (8433 to 12586)	16.4 (13.3 to 19.8)	-8.29 (-8.92 to -7.66)
Tropical Latin America	108760 (96938 to 123091)	202.9 (180.8 to 229.6)	10972 (8980 to 13333)	21.9 (17.9 to 26.6)	-7.34 (-7.64 to -7.04)
North Africa and Middle East	681452 (538611 to 847154)	485.1 (383.4 to 603)	154498 (119467 to 204086)	84.3 (65.2 to 111.3)	-5.22 (-5.52 to -4.91)
South Asia	8173463 (6639311 to 9841901)	1886.1 (1532 to 2271.1)	1461670 (1217873 to 1722256)	288.3 (240.2 to 339.7)	-5.86 (-5.95 to -5.78)
Central Sub-Saharan Africa	1995172 (1437679 to 2594802)	7886.5 (5682.8 to 10256.7)	836340 (547576 to 1278083)	1425.2 (933.1 to 2178.0)	-5.05 (-5.69 to -4.4)
Eastern Sub-Saharan Africa	4966907 (3929063 to 6016571)	5484.0 (4338.1 to 6643)	1516737 (1127159 to 1999744)	850.0 (631.7 to 1120.7)	-5.83 (-5.96 to -5.71)
Southern Sub-Saharan Africa	457529 (377838 to 581549)	2211.4 (1826.2 to 2810.9)	252989 (208357 to 307226)	1051.2 (865.8 to 1276.6)	-1.96 (-2.45 to -1.47)
Western Sub-Saharan Africa	3055646 (2295848 to 3843550)	3477.1 (2612.5 to 4373.6)	1817142 (1209776 to 2716545)	846.1 (563.3 to 1264.9)	-4.39 (-4.75 to -4.03)

Table S2. The incidence of drug-susceptible tuberculosis in children aged 0-14 years in 1990 and 2021 and estimated annual percentage change from 1990 to 2021. (Generated from data available at https://ghdx.healthdata.org/gbd-results-tool)

	Incidence				

	Cases (n), 1990	ASR per 100 000, 1990	Cases (n), 2021	ASR per 100 000, 2021	EAPC 1990-2021
Overall	1420310 (1107523 to 1777803)	81.7 (63.7 to 102.2)	725593 (568127 to 910846)	36.1 (28.2 to 45.3)	-2.70 (-2.82 to -2.59)
**Sex**					
Male	569561 (444762 to 712927)	63.7 (49.8 to 79.8)	302080 (236344 to 381168)	29.1 (22.8 to 36.7)	-2.56 (-2.64 to -2.48)
Female	850749 (661034 to 1062844)	100.6 (78.2 to 125.7)	423513 (332828 to 527416)	43.5 (34.2 to 54.2)	-2.80 (-2.95 to -2.65)
**SDI level**					
Low SDI	380429 (304488 to 467648)	166.2 (133.0 to 204.3)	311113 (245518 to 389007)	67.6 (53.3 to 84.5)	-3.03 (-3.20 to -2.86)
Low-middle SDI	550381 (412295 to 700220)	116.6 (87.3 to 148.3)	238197 (185027 to 303012)	41.1 (31.9 to 52.3)	-3.58 (-3.72 to -3.44)
Middle SDI	386023 (301045 to 484652)	66.9 (52.2 to 84.0)	155000 (119920 to 197364)	27.3 (21.2 to 34.8)	-2.91 (-2.98 to -2.84)
High-middle SDI	90637 (70614 to 114807)	33.1 (25.8 to 42.0)	17201 (13101 to 22212)	7.5 (5.7 to 9.6)	-4.76 (-4.85 to -4.67)
High SDI	12123 (9450 to 15264)	6.5 (5.1 to 8.2)	3626 (2682 to 4811)	2.1 (1.6 to 2.8)	-3.72 (-3.90 to -3.53)
**Region**					
East Asia	172939 (134285 to 215919)	52.4 (40.7 to 65.5)	26327 (20041 to 33870)	9.8 (7.5 to 12.7)	-5.12 (-5.28 to -4.95)
Southeast Asia	178686 (137998 to 226435)	104.6 (80.8 to 132.6)	94867 (73625 to 120094)	54.9 (42.6 to 69.6)	-2.21 (-2.48 to -1.94)
Oceania	1430 (1069 to 1858)	53.4 (39.9 to 69.3)	2116 (1600 to 2748)	41.6 (31.5 to 54.1)	-0.75 (-0.90 to -0.6)
Central Asia	8273 (6325 to 10543)	33.1 (25.3 to 42.2)	3026 (2265 to 3987)	10.9 (8.2 to 14.4)	-3.38 (-3.57 to -3.19)

Central Europe	3231 (2421 to 4220)	11.0 (8.2 to 14.3)	660 (498 to 866)	3.7 (2.8 to 4.9)	-3.46 (-3.53 to -3.39)
Eastern Europe	12039 (8911 to 16148)	23.4 (17.3 to 31.4)	2241 (1453 to 3268)	6.3 (4.1 to 9.2)	-2.98 (-3.37 to -2.59)
High-income Asia Pacific	1764 (1449 to 2185)	5.0 (4.1 to 6.2)	189 (135 to 259)	0.8 (0.6 to 1.2)	-5.60 (-5.79 to -5.40)
Australasia	159 (118 to 214)	3.5 (2.6 to 4.7)	87 (66 to 114)	1.5 (1.1 to 2.0)	-3.08 (-3.24 to -2.92)
Western Europe	3942 (2911 to 5317)	5.6 (4.1 to 7.5)	1699 (1210 to 2356)	2.5 (1.8 to 3.5)	-2.19 (-2.40 to -1.98)
Southern Latin America	1612 (1205 to 2083)	10.8 (8.1 to 14.0)	730 (538 to 970)	5.0 (3.7 to 6.7)	-2.83 (-3.05 to -2.61)
High-income North America	474 (343 to 639)	0.8 (0.6 to 1.0)	509 (379 to 673)	0.8 (0.6 to 1.0)	1.15 (0.64 to 1.66)
Caribbean	4745 (3735 to 5928)	41.6 (32.7 to 51.9)	2391 (1833 to 3026)	20.8 (15.9 to 26.3)	-2.25 (-2.41 to -2.09)
Andean Latin America	12554 (9500 to 16067)	84.5 (64.0 to 108.2)	2934 (2247 to 3897)	16.2 (12.4 to 21.5)	-5.76 (-6.14 to -5.38)
Central Latin America	8671 (6670 to 10829)	13.5 (10.4 to 16.8)	2586 (1931 to 3335)	4.1 (3.0 to 5.3)	-4.45 (-4.74 to -4.16)
Tropical Latin America	8295 (6323 to 10695)	15.5 (11.8 to 19.9)	2912 (2148 to 3807)	5.8 (4.3 to 7.6)	-4.62 (-5.34 to -3.89)
North Africa and Middle East	54282 (42455 to 67557)	38.6 (30.2 to 48.1)	21319 (16425 to 27286)	11.6 (9.0 to 14.9)	-3.70 (-3.96 to -3.43)
South Asia	486771 (352962 to 642188)	112.3 (81.4 to 148.2)	180544 (134574 to 236310)	35.6 (26.5 to 46.6)	-4.14 (-4.31 to -3.97)
Central Sub-Saharan Africa	72794 (57895 to 90449)	287.7 (228.8 to 357.5)	83279 (65418 to 103485)	141.9 (111.5 to 176.4)	-2.23 (-2.52 to -1.93)
Eastern Sub-Saharan Africa	163723 (132120 to 198995)	180.8 (145.9 to 219.7)	131625 (101881 to 168436)	73.8 (57.1 to 94.4)	-3.17 (-3.35 to -2.99)
Southern Sub-Saharan Africa	68179 (54752 to 84342)	329.5 (264.6 to 407.7)	38018 (28690 to 49038)	158.0 (119.2 to 203.8)	-2.08 (-2.38 to -1.79)
Western Sub-Saharan Africa	155747 (124838 to 191493)	177.2 (142.1 to 217.9)	127533 (102955 to 158993)	59.4 (47.9 to 74.0)	-3.49 (-3.77 to -3.22)

Table S3. The disability adjusted life years (DALYs) of drug-susceptible tuberculosis in children aged 0-14 years in 1990 and 2021 and estimated annual percentage change from 1990 to 2021. (Generated from data available at https://ghdx.healthdata.org/gbd-results-tool)

	DALYs				
	Cases (n), 1990	ASR per 100 000, 1990	Cases (n), 2021	ASR per 100 000, 2021	EAPC 1990-2021
Overall	24703150 (20751972 to 28631645)	1420.4 (1193.2 to 1646.3)	6093957 (4702774 to 7595268)	302.9 (233.8 to 377.5)	-4.59 (-4.80 to -4.39)
**Sex**					
Male	10427610 (7171103 to 12734652)	1167.1 (802.6 to 1425.3)	2708249 (1985373 to 3643023)	260.9 (191.2 to 350.9)	-4.44 (-4.66 to -4.23)
Female	14275540 (11945522 to 17290740)	1688.1 (1412.6 to 2044.6)	3385708 (2723258 to 4116880)	347.7 (279.7 to 422.8)	-4.71 (-4.91 to -4.50)
**SDI level**					
Low SDI	10084553 (8164043 to 11971951)	4405.4 (3566.4 to 5229.9)	3732306 (2673951 to 4852611)	811.0 (581.0 to 1054.4)	-5.22 (-5.44 to -5.01)
Low-middle SDI	9506287 (7778806 to	2013.6 (1647.7 to	1764592 (1424030 to	304.3 (245.6 to	-5.79 (-5.92 to -5.66)

	11274051)	2388.0)	2130304)	367.4)	
Middle SDI	4417934 (3901855 to 4895365)	765.4 (676.0 to 848.1)	553032 (469563 to 649034)	97.6 (82.8 to 114.5)	-6.06 (-6.20 to -5.92)
High-middle SDI	627445 (535838 to 717769)	229.3 (195.8 to 262.3)	36654 (30194 to 42928)	15.9 (13.1 to 18.6)	-8.10 (-8.22 to -7.98)
High SDI	54029 (46071 to 63579)	29.1 (24.8 to 34.2)	3531 (2832 to 4413)	2.0 (1.6 to 2.6)	-8.22 (-8.58 to -7.86)
**Region**					
East Asia	1850532 (1520509 to 2225723)	561.0 (461.0 to 674.8)	43985 (34669 to 53610)	16.5 (13.0 to 20.1)	-10.32 (-10.46 to -10.18)
Southeast Asia	2797787 (2133341 to 3292014)	1638.6 (1249.4 to 1928.0)	415913 (348176 to 492082)	240.9 (201.7 to 285.0)	-5.95 (-6.05 to -5.86)
Oceania	23863 (15295 to 37299)	890.5 (570.7 to 1391.8)	23671 (14254 to 34089)	465.9 (280.5 to 670.9)	-1.83 (-2.25 to -1.40)
Central Asia	134781 (121638 to 149438)	539.3 (486.7 to 598.0)	20343 (13365 to 28885)	73.5 (48.3 to 104.4)	-6.73 (-7.09 to -6.37)
Central Europe	14763 (13660 to 15824)	50.1 (46.3 to 53.7)	1275 (1058 to 1507)	7.2 (6.0 to 8.5)	-6.45 (-6.81 to -6.09)
Eastern Europe	30309 (27794 to 32824)	58.9 (54.0 to 63.8)	2131 (1433 to 2843)	6.0 (4.0 to 8.0)	-6.18 (-7.10 to -5.26)
High-income Asia Pacific	16108 (13194 to 19566)	45.8 (37.5 to 55.6)	401 (343 to 470)	1.8 (1.5 to 2.1)	-9.99 (-10.43 to -9.54)
Australasia	216 (172 to 278)	4.7 (3.8 to 6.1)	78 (53 to 111)	1.4 (0.9 to 1.9)	-3.91 (-4.04 to -3.77)
Western Europe	3829 (3366 to 4407)	5.4 (4.7 to 6.2)	827 (610 to 1108)	1.2 (0.9 to 1.6)	-4.37 (-4.47 to -4.26)
Southern Latin America	13840 (13079 to 14739)	92.7 (87.6 to 98.7)	1434 (1231 to 1675)	9.9 (8.5 to 11.6)	-6.68 (-7.08 to -6.27)
High-income North America	2352 (2151 to 2558)	3.8 (3.5 to 4.1)	711 (550 to 911)	1.1 (0.8 to 1.4)	-3.28 (-3.83 to -2.73)
Caribbean	90927 (73642 to 116879)	796.7 (645.3 to 1024.1)	24228 (16610 to 39017)	210.6 (144.4 to 339.1)	-3.75 (-4.18 to -3.32)
Andean Latin America	220004 (179726 to	1481.3 (1210.1 to	12373 (9357 to 16009)	68.4 (51.7 to 88.5)	-9.63 (-10.04 to

	260005)	1750.6)			-9.22)
Central Latin America	126190 (118353 to 135195)	196.0 (183.8 to 210.0)	9743 (8006 to 11940)	15.3 (12.6 to 18.8)	-8.42 (-9.09 to -7.75)
Tropical Latin America	108641 (96889 to 122975)	202.6 (180.7 to 229.4)	10263 (7972 to 12801)	20.4 (15.9 to 25.5)	-7.53 (-7.84 to -7.21)
North Africa and Middle East	678226 (536074 to 843547)	482.8 (381.6 to 600.5)	142768 (104502 to 192421)	77.9 (57.0 to 105.0)	-5.47 (-5.72 to -5.22)
South Asia	8157382 (6620811 to 9821314)	1882.3 (1527.8 to 2266.3)	1277077 (996026 to 1566464)	251.9 (196.4 to 309.0)	-6.28 (-6.36 to -6.21)
Central Sub-Saharan Africa	1983070 (1421378 to 2565046)	7838.6 (5618.4 to 10139.1)	795667 (522075 to 1235449)	1355.9 (889.7 to 2105.3)	-5.16 (-5.79 to -4.52)
Eastern Sub-Saharan Africa	4957203 (3924093 to 6009055)	5473.3 (4332.6 to 6634.7)	1386343 (1029767 to 1837465)	777.0 (577.1 to 1029.8)	-6.08 (-6.20 to -5.97)
Southern Sub-Saharan Africa	453644 (375014 to 574376)	2192.6 (1812.6 to 2776.2)	232228 (182958 to 289297)	965.0 (760.2 to 1202.1)	-2.19 (-2.65 to -1.74)
Western Sub-Saharan Africa	3039485 (2285658 to 3828753)	3458.7 (2600.9 to 4356.8)	1692498 (1124468 to 2526376)	788.1 (523.6 to 1176.4)	-4.58 (-4.90 to -4.26)

Table S4. The incidence of multidrug-resistant tuberculosis in children aged 0-14 years in 1990 and 2021 and estimated annual percentage change from 1990 to 2021. (Generated from data available at https://ghdx.healthdata.org/gbd-results-tool)

	Incidence				
	Cases (n), 1990	ASR per 100 000, 1990	Cases (n), 2021	ASR per 100 000, 2021	EAPC 1990-2021
Overall	8086 (3542 to 19038)	0.5 (0.2 to 1.1)	32515 (20968 to 51288)	1.6 (1.0 to 2.5)	1.18 (-0.16 to 2.54)
**Sex**					
Male	3800 (1619 to 9194)	0.4 (0.2 to 1.0)	13546 (8563 to 21718)	1.3 (0.8 to 2.1)	0.88 (-0.37 to 2.16)
Female	4286 (1931 to 9790)	0.5 (0.2 to 1.2)	18968 (12178 to 29628)	1.9 (1.3 to 3.0)	1.41 (0.00 to 2.84)
**SDI level**					
Low SDI	874 (494 to 1574)	0.4 (0.2 to 0.7)	12087 (7218 to 18796)	2.6 (1.6 to 4.1)	2.77 (0.97 to 4.61)
Low-middle SDI	669 (325 to 1472)	0.1 (0.1 to 0.3)	12217 (6110 to 22486)	2.1 (1.1 to 3.9)	4.33 (2.04 to 6.67)
Middle SDI	4622 (1532 to 12192)	0.8 (0.3 to 2.1)	6500 (3464 to 11030)	1.1 (0.6 to 1.9)	-1.68 (-2.71 to -0.63)
High-middle SDI	1793 (478 to 5072)	0.7 (0.2 to 1.9)	1609 (931 to 2583)	0.7 (0.4 to 1.1)	-2.57 (-3.68 to -1.44)
High SDI	125 (68 to 235)	0.1 (0.0 to 0.1)	89 (55 to 148)	0.1 (0.0 to 0.1)	-3.88 (-4.80 to -2.96)
**Region**					

East Asia	5564 (1426 to 16647)	1.7 (0.4 to 5.0)	1147 (290 to 3126)	0.4 (0.1 to 1.2)	-7.93 (-9.12 to -6.72)
Southeast Asia	383 (132 to 987)	0.2 (0.1 to 0.6)	1922 (862 to 3791)	1.1 (0.5 to 2.2)	0.32 (-1.25 to 1.91)
Oceania	1 (0 to 2)	0.0 (0.0 to 0.1)	77 (22 to 173)	1.5 (0.4 to 3.4)	11.99 (10.49 to 13.52)
Central Asia	14 (5 to 35)	0.1 (0.0 to 0.1)	866 (544 to 1388)	3.1 (2.0 to 5.0)	9.76 (6.48 to 13.13)
Central Europe	22 (9 to 50)	0.1 (0.0 to 0.2)	14 (6 to 27)	0.1 (0.0 to 0.2)	-1.33 (-2.31 to -0.34)
Eastern Europe	140 (55 to 371)	0.3 (0.1 to 0.7)	982 (528 to 1656)	2.8 (1.5 to 4.7)	5.57 (3.69 to 7.49)
High-income Asia Pacific	10 (3 to 29)	0.0 (0.0 to 0.1)	2 (1 to 6)	0.0 (0.0 to 0.0)	-6.75 (-8.42 to -5.04)
Australasia	1 (0 to 2)	0.0 (0.0 to 0.0)	2 (1 to 5)	0.0 (0.0 to 0.1)	2.63 (2.29 to 2.97)
Western Europe	29 (16 to 51)	0.0 (0.0 to 0.1)	37 (22 to 60)	0.1 (0.0 to 0.1)	0.19 (-0.70 to 1.09)
Southern Latin America	6 (1 to 17)	0.0 (0.0 to 0.1)	9 (2 to 31)	0.1 (0.0 to 0.2)	-1.45 (-3.20 to 0.34)
High-income North America	13 (7 to 23)	0.0 (0.0 to 0.0)	8 (3 to 17)	0.0 (0.0 to 0.0)	-0.43 (-1.47 to 0.62)
Caribbean	17 (5 to 46)	0.1 (0.0 to 0.4)	10 (3 to 29)	0.1 (0.0 to 0.3)	-4.87 (-5.98 to -3.75)
Andean Latin America	107 (32 to 264)	0.7 (0.2 to 1.8)	215 (95 to 430)	1.2 (0.5 to 2.4)	-1.92 (-3.46 to -0.35)
Central Latin America	13 (6 to 27)	0.0 (0.0 to 0.0)	75 (33 to 149)	0.1 (0.1 to 0.2)	0.14 (-2.04 to 2.37)
Tropical Latin America	5 (1 to 16)	0.0 (0.0 to 0.0)	98 (21 to 296)	0.2 (0.0 to 0.6)	3.74 (0.92 to 6.64)
North Africa and Middle East	145 (64 to 289)	0.1 (0.0 to 0.2)	681 (307 to 1564)	0.4 (0.2 to 0.9)	2.06 (-0.10 to 4.27)
South Asia	439 (113 to 1330)	0.1 (0.0 to 0.3)	13226 (4816 to 28148)	2.6 (0.9 to 5.6)	5.71 (3.10 to 8.38)
Central Sub-Saharan Africa	234 (59 to 785)	0.9 (0.2 to 3.1)	1913 (603 to 4866)	3.3 (1.0 to 8.3)	1.09 (-0.25 to 2.44)
Eastern Sub-Saharan Africa	181 (72 to 417)	0.2 (0.1 to 0.5)	5290 (3110 to 8557)	3.0 (1.7 to 4.8)	4.00 (1.79 to 6.25)
Southern Sub-Saharan Africa	286 (59 to 830)	1.4 (0.3 to 4.0)	1513 (688 to 3274)	6.3 (2.9 to 13.6)	3.05 (1.10 to 5.04)
Western Sub-Saharan Africa	480 (230 to 885)	0.5 (0.3 to 1.0)	4428 (1986 to 9775)	2.1 (0.9 to 4.6)	1.24 (-0.53 to 3.05)

Table S5. The disability adjusted life years (DALYs) of multidrug-resistant tuberculosis in children aged 0-14 years in 1990 and 2021 and estimated annual percentage change from 1990 to 2021. (Generated from data available at https://ghdx.healthdata.org/gbd-results-tool)

	DALYs				
	Cases (n), 1990	ASR per 100 000, 1990	Cases (n), 2021	ASR per 100 000, 2021	EAPC 1990-2021
Overall	189110 (69056 to 436990)	10.9 (4.0 to 25.1)	530364 (235225 to 1002206)	26.4 (11.7 to 49.8)	0.51 (-1.05 to 2.09)
**Sex**					
Male	88669 (32032 to 213825)	9.9 (3.6 to 23.9)	231866 (94019 to 462832)	22.3 (9.1 to 44.6)	0.30 (-1.21 to 1.83)
Female	100441 (36734 to 233386)	11.9 (4.3 to 27.6)	298498 (132318 to 567577)	30.7 (13.6 to 58.3)	0.68 (-0.92 to 2.31)
**SDI level**					
Low SDI	38524 (13592 to 89215)	16.8 (5.9 to 39.0)	303575 (125140 to 631806)	66 (27.2 to 137.3)	1.31 (-0.70 to 3.36)
Low-middle SDI	23589 (8580 to 56850)	5.0 (1.8 to 12.0)	182112 (65508 to	31.4 (11.3 to 63.3)	2.31 (-0.09 to 4.76)

			367132)		
Middle SDI	104814 (28250 to 298912)	18.2 (4.9 to 51.8)	41055 (17840 to 77869)	7.2 (3.1 to 13.7)	-4.97 (-5.90 to -4.02)
High-middle SDI	21213 (5263 to 63687)	7.8 (1.9 to 23.3)	3255 (1773 to 5437)	1.4 (0.8 to 2.4)	-7.67 (-8.93 to -6.4)
High SDI	920 (409 to 1959)	0.5 (0.2 to 1.1)	136 (65 to 280)	0.1 (0.0 to 0.2)	-8.41 (-9.42 to -7.38)
**Region**					
East Asia	110251 (25335 to 326607)	33.4 (7.7 to 99.0)	3290 (782 to 8601)	1.2 (0.3 to 3.2)	-12.93 (-14.03 to -11.82)
Southeast Asia	11154 (2882 to 34077)	6.5 (1.7 to 20.0)	14483 (5310 to 31187)	8.4 (3.1 to 18.1)	-3.35 (-5.39 to -1.27)
Oceania	23 (4 to 85)	0.8 (0.1 to 3.2)	1747 (369 to 4832)	34.4 (7.3 to 95.1)	11.40 (9.64 to 13.19)
Central Asia	357 (100 to 972)	1.4 (0.4 to 3.9)	7909 (4092 to 12749)	28.6 (14.8 to 46.1)	6.22 (2.77 to 9.79)
Central Europe	214 (59 to 579)	0.7 (0.2 to 2.0)	57 (17 to 136)	0.3 (0.1 to 0.8)	-4.56 (-5.52 to -3.59)
Eastern Europe	731 (242 to 2006)	1.4 (0.5 to 3.9)	1119 (632 to 1582)	3.2 (1.8 to 4.5)	0.08 (-2.55 to 2.77)
High-income Asia Pacific	197 (36 to 686)	0.6 (0.1 to 1.9)	7 (2 to 24)	0.0 (0.0 to 0.1)	-12.04 (-13.38 to -10.67)
Australasia	2 (1 to 7)	0.0 (0.0 to 0.2)	3 (1 to 6)	0.0 (0.0 to 0.1)	0.33 (-0.20 to 0.87)
Western Europe	54 (24 to 98)	0.1 (0.0 to 0.1)	25 (13 to 44)	0.0 (0.0 to 0.1)	-3.20 (-3.76 to -2.64)
Southern Latin America	98 (23 to 287)	0.7 (0.2 to 1.9)	32 (7 to 103)	0.2 (0.0 to 0.7)	-5.66 (-6.91 to -4.4)
High-income North America	144 (62 to 308)	0.2 (0.1 to 0.5)	15 (5 to 40)	0.0 (0.0 to 0.1)	-6.61 (-7.96 to -5.24)
Caribbean	586 (114 to 1867)	5.1 (1.0 to 16.4)	198 (27 to 768)	1.7 (0.2 to 6.7)	-5.90 (-6.77 to -5.03)
Andean Latin America	3649 (818 to 11009)	24.6 (5.5 to 74.1)	1659 (596 to 3561)	9.2 (3.3 to 19.7)	-6.14 (-7.46 to -4.80)
Central Latin America	373 (116 to 874)	0.6 (0.2 to 1.4)	549 (189 to 1206)	0.9 (0.3 to 1.9)	-3.83 (-5.84 to -1.77)
Tropical Latin America	119 (15 to 512)	0.2 (0.0 to 1.0)	610 (126 to 1702)	1.2 (0.3 to 3.4)	0.83 (-1.93 to 3.68)
North Africa and Middle East	3226 (934 to 7587)	2.3 (0.7 to 5.4)	10904 (2494 to 32841)	5.9 (1.4 to 17.9)	1.19 (-1.28 to 3.71)
South Asia	16081 (3177 to 52142)	3.7 (0.7 to 12.0)	175553 (56903 to	34.6 (11.2 to 74.9)	3.59 (0.91 to 6.33)

			379967)		
Central Sub-Saharan Africa	12102 (2092 to 48967)	47.8 (8.3 to 193.6)	40123 (10185 to 128079)	68.4 (17.4 to 218.3)	-1.29 (-2.95 to 0.40)
Eastern Sub-Saharan Africa	9704 (2778 to 27375)	10.7 (3.1 to 30.2)	128637 (45698 to 279409)	72.1 (25.6 to 156.6)	2.11 (-0.23 to 4.50)
Southern Sub-Saharan Africa	3885 (647 to 12225)	18.8 (3.1 to 59.1)	20484 (7307 to 46220)	85.1 (30.4 to 192.1)	3.57 (1.90 to 5.28)
Western Sub-Saharan Africa	16162 (5188 to 38818)	18.4 (5.9 to 44.2)	122961 (37060 to 327219)	57.3 (17.3 to 152.4)	0.95 (-1.02 to 2.95)

Table S6. The change of age-standardised incidence rate of tuberculosis among children aged 0-14 years from 1990 to 2021 at national level.

**Countries**	**EAPC No. (95% CI)**
Afghanistan	-3.09 (-3.79 to -2.38)
Albania	-3.43 (-3.80 to -3.06)
Algeria	-4.44 (-4.91 to -3.96)
American Samoa	-3.14 (-3.64 to -2.63)
Andorra	-3.56 (-3.77 to -3.35)
Angola	-2.56 (-2.99 to -2.12)
Antigua and Barbuda	-2.56 (-2.93 to -2.18)
Argentina	-2.39 (-2.60 to -2.18)
Armenia	-2.82 (-3.15 to -2.48)
Australia	-3.03 (-3.18 to -2.89)
Austria	-2.49 (-2.65 to -2.33)
Azerbaijan	-3.31 (-3.55 to -3.06)
Bahamas	-3.24 (-3.57 to -2.92)
Bahrain	-3.17 (-3.41 to -2.93)
Bangladesh	-4.51 (-5.37 to -3.64)
Barbados	-2.64 (-3.24 to -2.05)
Belarus	-6.18 (-6.52 to -5.84)
Belgium	-1.56 (-1.73 to -1.39)
Belize	-4.76 (-4.99 to -4.54)
Benin	-3.45 (-3.63 to -3.27)
Bermuda	-3.39 (-4.06 to -2.70)
Bhutan	-3.45 (-4.22 to -2.66)
Bolivia (Plurinational State of)	-5.23 (-5.56 to -4.91)
Bosnia and Herzegovina	-3.15 (-3.70 to -2.61)
Botswana	-1.52 (-2.07 to -0.98)
Brazil	-4.60 (-5.02 to -4.19)
Brunei Darussalam	-3.06 (-3.44 to -2.68)
Bulgaria	0.12 (-0.29 to 0.53)
Burkina Faso	-3.26 (-3.50 to -3.01)
Burundi	-3.35 (-3.99 to -2.70)
Cabo Verde	-4.55 (-4.82 to -4.27)
Cambodia	-3.74 (-4.29 to -3.20)
Cameroon	-2.41 (-2.86 to -1.96)
Canada	-0.06 (-0.35 to 0.23)
Central African Republic	-1.35 (-1.82 to -0.88)
Chad	-2.49 (-2.74 to -2.23)
Chile	-5.34 (-5.64 to -5.05)
China	-5.64 (-5.80 to -5.49)
Colombia	-5.06 (-5.20 to -4.92)
Comoros	-3.56 (-4.12 to -3.00)
Congo	-2.49 (-3.06 to -1.92)

Cook Islands	-3.46 (-4.13 to -2.78)
Costa Rica	-4.95 (-5.20 to -4.71)
Côte d'Ivoire	-3.03 (-3.24 to -2.82)
Croatia	-4.72 (-5.14 to -4.29)
Cuba	-3.54 (-3.69 to -3.39)
Cyprus	-2.95 (-3.36 to -2.53)
Czechia	-4.01 (-4.48 to -3.55)
Democratic People's Republic of Korea	-2.00 (-2.30 to -1.70)
Democratic Republic of the Congo	-1.99 (-2.53 to -1.44)
Denmark	-3.71 (-4.00 to -3.42)
Djibouti	-3.42 (-3.99 to -2.84)
Dominica	-2.21 (-2.33 to -2.10)
Dominican Republic	-4.16 (-4.46 to -3.85)
Ecuador	-6.01 (-6.26 to -5.77)
Egypt	-4.49 (-4.81 to -4.17)
El Salvador	-5.01 (-5.23 to -4.78)
Equatorial Guinea	-4.71 (-5.21 to -4.20)
Eritrea	-2.54 (-2.93 to -2.14)
Estonia	-4.00 (-4.18 to -3.82)
Eswatini	-0.75 (-1.03 to -0.47)
Ethiopia	-4.03 (-4.55 to -3.51)
Fiji	-2.09 (-2.86 to -1.32)
Finland	-2.55 (-2.77 to -2.33)
France	-3.70 (-3.98 to -3.42)
Gabon	-1.46 (-1.93 to -0.99)
Gambia	-3.30 (-3.41 to -3.20)
Georgia	-0.25 (-0.75 to 0.25)
Germany	-2.44 (-2.64 to -2.23)
Ghana	-3.18 (-3.34 to -3.02)
Greece	-2.19 (-2.28 to -2.10)
Greenland	0.14 (-0.17 to 0.45)
Grenada	-2.64 (-3.30 to -1.97)
Guam	-1.53 (-2.25 to -0.80)
Guatemala	-4.52 (-4.70 to -4.33)
Guinea	-2.89 (-3.13 to -2.65)
Guinea-Bissau	-3.20 (-3.67 to -2.72)
Guyana	-3.70 (-3.85 to -3.56)
Haiti	-2.87 (-3.20 to -2.54)
Honduras	-4.78 (-5.26 to -4.30)
Hungary	-5.78 (-6.10 to -5.46)
Iceland	-1.65 (-1.95 to -1.34)
India	-4.19 (-4.55 to -3.82)
Indonesia	-3.53 (-3.79 to -3.28)
Iran (Islamic Republic of)	-2.67 (-3.15 to -2.19)

Iraq	-5.07 (-5.57 to -4.57)
Ireland	-3.38 (-3.57 to -3.19)
Israel	-3.63 (-3.78 to -3.48)
Italy	-2.22 (-2.35 to -2.09)
Jamaica	-2.81 (-2.91 to -2.71)
Japan	-4.35 (-4.72 to -3.98)
Jordan	-3.85 (-4.33 to -3.37)
Kazakhstan	-4.74 (-5.38 to -4.10)
Kenya	-3.16 (-3.72 to -2.59)
Kiribati	-1.29 (-1.86 to -0.71)
Kuwait	-5.04 (-5.41 to -4.66)
Kyrgyzstan	-0.36 (-0.62 to -0.09)
Lao People's Democratic Republic	-4.88 (-5.33 to -4.42)
Latvia	-3.06 (-3.41 to -2.71)
Lebanon	-4.71 (-5.19 to -4.24)
Lesotho	-0.03 (-0.29 to 0.23)
Liberia	-5.59 (-5.83 to -5.34)
Libya	-2.31 (-2.68 to -1.93)
Lithuania	-1.31 (-1.70 to -0.92)
Luxembourg	-1.76 (-1.90 to -1.62)
Madagascar	-3.12 (-3.73 to -2.52)
Malawi	-2.94 (-3.81 to -2.06)
Malaysia	-3.20 (-3.41 to -2.98)
Maldives	-5.13 (-5.61 to -4.63)
Mali	-3.76 (-3.87 to -3.64)
Malta	-1.59 (-1.70 to -1.49)
Marshall Islands	-0.83 (-1.63 to -0.02)
Mauritania	-4.12 (-4.33 to -3.91)
Mauritius	-1.52 (-1.85 to -1.19)
Mexico	-4.15 (-4.51 to -3.79)
Micronesia (Federated States of)	-2.03 (-2.74 to -1.32)
Monaco	-3.33 (-3.71 to -2.95)
Mongolia	-4.26 (-4.56 to -3.96)
Montenegro	-3.31 (-3.48 to -3.13)
Morocco	-4.29 (-4.67 to -3.91)
Mozambique	-2.12 (-2.76 to -1.47)
Myanmar	-4.72 (-4.87 to -4.57)
Namibia	-1.85 (-2.08 to -1.61)
Nauru	-0.72 (-1.52 to 0.09)
Nepal	-4.69 (-5.05 to -4.33)
Netherlands	-4.85 (-5.23 to -4.46)
New Zealand	-2.82 (-3.02 to -2.62)
Nicaragua	-5.72 (-5.91 to -5.52)
Niger	-3.93 (-4.23 to -3.62)

Nigeria	-3.64 (-3.97 to -3.31)
Niue	-1.36 (-2.06 to -0.66)
North Macedonia	-4.50 (-4.86 to -4.15)
Northern Mariana Islands	-1.03 (-1.59 to -0.47)
Norway	-2.04 (-2.25 to -1.84)
Oman	-4.10 (-4.47 to -3.74)
Pakistan	-2.72 (-3.60 to -1.82)
Palau	-1.31 (-1.90 to -0.72)
Palestine	-3.69 (-4.03 to -3.34)
Panama	-3.98 (-4.26 to -3.71)
Papua New Guinea	-0.65 (-1.16 to -0.14)
Paraguay	-2.54 (-2.66 to -2.43)
Peru	-5.58 (-5.93 to -5.24)
Philippines	4.44 (3.56 to 5.32)
Poland	-2.03 (-2.45 to -1.60)
Portugal	-5.24 (-5.39 to -5.09)
Puerto Rico	-4.10 (-4.25 to -3.95)
Qatar	-4.87 (-5.37 to -4.37)
Republic of Korea	-5.71 (-6.04 to -5.38)
Republic of Moldova	-0.71 (-0.97 to -0.45)
Romania	-3.56 (-3.73 to -3.40)
Russian Federation	-1.63 (-1.97 to -1.28)
Rwanda	-3.79 (-4.22 to -3.36)
Saint Kitts and Nevis	-3.55 (-3.68 to -3.43)
Saint Lucia	-3.65 (-3.94 to -3.36)
Saint Vincent and the Grenadines	-3.55 (-4.06 to -3.04)
Samoa	-1.76 (-2.44 to -1.08)
San Marino	-3.49 (-4.60 to -2.36)
Sao Tome and Principe	-4.71 (-4.79 to -4.63)
Saudi Arabia	-6.17 (-6.85 to -5.47)
Senegal	-3.89 (-4.08 to -3.69)
Serbia	-3.17 (-3.31 to -3.03)
Seychelles	-1.73 (-2.13 to -1.34)
Sierra Leone	-3.38 (-3.69 to -3.06)
Singapore	-4.65 (-4.92 to -4.38)
Slovakia	-3.71 (-4.09 to -3.34)
Slovenia	-3.68 (-4.04 to -3.32)
Solomon Islands	-1.06 (-2.35 to 0.24)
Somalia	-1.58 (-2.20 to -0.95)
South Africa	-2.36 (-2.81 to -1.91)
South Sudan	-1.27 (-1.82 to -0.71)
Spain	-4.88 (-5.24 to -4.52)
Sri Lanka	-2.68 (-3.07 to -2.29)
Sudan	-4.02 (-4.53 to -3.51)

Suriname	-3.46 (-3.81 to -3.10)
Sweden	1.21 (1.06 to 1.36)
Switzerland	-1.87 (-2.07 to -1.67)
Syrian Arab Republic	-3.76 (-4.39 to -3.12)
Taiwan (Province of China)	-4.95 (-5.88 to -4.01)
Tajikistan	-3.53 (-3.89 to -3.17)
Thailand	-1.20 (-1.48 to -0.92)
Timor-Leste	-2.28 (-2.78 to -1.79)
Togo	-3.12 (-3.36 to -2.88)
Tokelau	-2.62 (-3.48 to -1.75)
Tonga	-1.45 (-2.17 to -0.73)
Trinidad and Tobago	-3.90 (-4.01 to -3.78)
Tunisia	-5.12 (-5.38 to -4.86)
Turkey	-6.82 (-6.99 to -6.65)
Turkmenistan	-3.04 (-3.34 to -2.74)
Tuvalu	-2.46 (-3.28 to -1.63)
Uganda	-2.24 (-2.72 to -1.75)
Ukraine	-0.58 (-1.01 to -0.14)
United Arab Emirates	-5.01 (-5.27 to -4.74)
United Kingdom	0.35 (-0.17 to 0.87)
United Republic of Tanzania	-3.85 (-4.59 to -3.10)
United States of America	1.47 (0.98 to 1.97)
United States Virgin Islands	-4.29 (-4.74 to -3.82)
Uruguay	-1.37 (-1.55 to -1.19)
Uzbekistan	0.03 (-0.38 to 0.44)
Vanuatu	-0.04 (-0.80 to 0.72)
Venezuela (Bolivarian Republic of)	-3.32 (-3.51 to -3.14)
Viet Nam	-3.25 (-3.76 to -2.73)
Yemen	-3.88 (-4.33 to -3.42)
Zambia	-3.11 (-3.86 to -2.36)
Zimbabwe	-0.82 (-1.21 to -0.43)

Table S7. The change of age-standardised DALYs rate of tuberculosis among children aged 0-14 years from 1990 to 2021 at national level.

Countries	EAPC No. (95% CI)
Afghanistan	-5.69 (-6.01 to -5.37)
Albania	-8.13 (-8.53 to -7.73)
Algeria	-6.71 (-6.99 to -6.43)
American Samoa	-4.54 (-4.85 to -4.22)
Andorra	-5.62 (-6.06 to -5.19)
Angola	-6.50 (-6.94 to -6.06)
Antigua and Barbuda	-4.08 (-4.24 to -3.92)
Argentina	-6.57 (-6.82 to -6.33)
Armenia	-5.77 (-6.58 to -4.95)
Australia	-3.67 (-3.87 to -3.46)
Austria	-4.24 (-4.45 to -4.03)
Azerbaijan	-5.98 (-6.22 to -5.74)
Bahamas	-5.96 (-6.25 to -5.67)
Bahrain	-5.58 (-5.86 to -5.31)
Bangladesh	-8.62 (-9.46 to -7.78)
Barbados	-4.76 (-5.03 to -4.50)
Belarus	-4.60 (-5.23 to -3.97)
Belgium	-3.47 (-3.59 to -3.34)
Belize	-7.69 (-7.99 to -7.39)
Benin	-4.96 (-5.12 to -4.79)
Bermuda	-4.11 (-4.84 to -3.37)
Bhutan	-7.19 (-7.73 to -6.65)
Bolivia (Plurinational State of)	-8.84 (-9.03 to -8.65)
Bosnia and Herzegovina	-5.90 (-6.48 to -5.32)
Botswana	-1.74 (-1.99 to -1.48)
Brazil	-7.54 (-7.76 to -7.32)
Brunei Darussalam	-4.92 (-5.48 to -4.35)
Bulgaria	-6.20 (-6.50 to -5.91)
Burkina Faso	-3.07 (-3.19 to -2.96)
Burundi	-5.24 (-5.78 to -4.69)
Cabo Verde	-8.78 (-9.30 to -8.26)
Cambodia	-7.75 (-8.00 to -7.50)
Cameroon	-3.36 (-3.88 to -2.85)
Canada	-3.85 (-4.20 to -3.50)
Central African Republic	-2.26 (-2.60 to -1.91)
Chad	-2.61 (-2.94 to -2.28)
Chile	-6.87 (-7.09 to -6.65)
China	-11.13 (-11.27 to -10.99)
Colombia	-7.03 (-7.33 to -6.72)
Comoros	-5.65 (-6.18 to -5.11)
Congo	-5.96 (-6.42 to -5.50)

Cook Islands	-7.76 (-8.16 to -7.37)
Costa Rica	-7.21 (-7.49 to -6.94)
Côte d'Ivoire	-3.58 (-3.91 to -3.24)
Croatia	-7.06 (-7.28 to -6.84)
Cuba	-5.46 (-5.73 to -5.19)
Cyprus	-5.77 (-6.12 to -5.41)
Czechia	-6.80 (-7.15 to -6.45)
Democratic People's Republic of Korea	-5.56 (-5.78 to -5.34)
Democratic Republic of the Congo	-4.82 (-5.35 to -4.28)
Denmark	-4.49 (-4.77 to -4.22)
Djibouti	-4.93 (-5.51 to -4.34)
Dominica	-2.29 (-2.66 to -1.91)
Dominican Republic	-7.51 (-7.83 to -7.20)
Ecuador	-9.19 (-9.39 to -8.99)
Egypt	-5.79 (-6.11 to -5.46)
El Salvador	-9.26 (-9.68 to -8.83)
Equatorial Guinea	-10.78 (-11.19 to -10.36)
Eritrea	-4.00 (-4.30 to -3.71)
Estonia	-6.42 (-6.99 to -5.85)
Eswatini	-1.59 (-1.99 to -1.18)
Ethiopia	-8.63 (-9.06 to -8.19)
Fiji	-2.07 (-2.55 to -1.59)
Finland	-4.90 (-5.42 to -4.38)
France	-5.58 (-6.04 to -5.12)
Gabon	-4.43 (-4.77 to -4.09)
Gambia	-4.55 (-4.86 to -4.25)
Georgia	-6.00 (-6.50 to -5.50)
Germany	-3.99 (-4.24 to -3.74)
Ghana	-4.28 (-4.60 to -3.95)
Greece	-3.97 (-4.21 to -3.74)
Greenland	-7.26 (-8.20 to -6.31)
Grenada	-5.19 (-5.35 to -5.04)
Guam	-2.65 (-3.13 to -2.16)
Guatemala	-10.76 (-10.94 to -10.58)
Guinea	-4.70 (-4.91 to -4.50)
Guinea-Bissau	-5.11 (-5.57 to -4.65)
Guyana	-5.17 (-5.54 to -4.79)
Haiti	-4.60 (-4.79 to -4.41)
Honduras	-8.68 (-8.88 to -8.47)
Hungary	-7.51 (-7.79 to -7.23)
Iceland	-4.48 (-5.35 to -3.60)
India	-6.50 (-6.78 to -6.22)
Indonesia	-5.77 (-5.94 to -5.61)
Iran (Islamic Republic of)	-6.49 (-6.90 to -6.08)

Iraq	-8.09 (-8.45 to -7.72)
Ireland	-4.80 (-5.08 to -4.51)
Israel	-5.35 (-5.60 to -5.10)
Italy	-4.04 (-4.31 to -3.76)
Jamaica	-6.36 (-6.83 to -5.89)
Japan	-5.19 (-5.52 to -4.86)
Jordan	-6.23 (-6.50 to -5.96)
Kazakhstan	-7.55 (-8.24 to -6.85)
Kenya	-4.47 (-5.20 to -3.74)
Kiribati	-3.83 (-4.08 to -3.59)
Kuwait	-4.71 (-5.18 to -4.24)
Kyrgyzstan	-5.31 (-5.78 to -4.84)
Lao People's Democratic Republic	-7.40 (-7.77 to -7.03)
Latvia	-5.61 (-6.18 to -5.04)
Lebanon	-7.97 (-8.35 to -7.60)
Lesotho	0.06 (-0.30 to 0.42)
Liberia	-8.92 (-9.25 to -8.60)
Libya	-2.36 (-2.79 to -1.91)
Lithuania	-5.42 (-5.98 to -4.85)
Luxembourg	-3.76 (-3.98 to -3.54)
Madagascar	-4.85 (-5.29 to -4.40)
Malawi	-7.47 (-8.08 to -6.85)
Malaysia	-6.04 (-6.34 to -5.75)
Maldives	-10.14 (-10.62 to -9.66)
Mali	-5.40 (-5.56 to -5.23)
Malta	-2.45 (-2.59 to -2.32)
Marshall Islands	-2.33 (-2.91 to -1.74)
Mauritania	-6.03 (-6.35 to -5.70)
Mauritius	-4.44 (-4.63 to -4.25)
Mexico	-8.44 (-9.00 to -7.87)
Micronesia (Federated States of)	-4.94 (-5.33 to -4.55)
Monaco	-5.04 (-5.54 to -4.55)
Mongolia	-8.00 (-8.23 to -7.77)
Montenegro	-7.09 (-7.52 to -6.66)
Morocco	-7.70 (-7.89 to -7.50)
Mozambique	-5.77 (-6.29 to -5.24)
Myanmar	-8.12 (-8.48 to -7.75)
Namibia	-2.68 (-2.93 to -2.42)
Nauru	-2.32 (-3.13 to -1.49)
Nepal	-9.03 (-9.29 to -8.77)
Netherlands	-5.27 (-5.66 to -4.88)
New Zealand	-3.96 (-4.36 to -3.55)
Nicaragua	-9.33 (-9.54 to -9.11)
Niger	-6.23 (-6.75 to -5.71)

Nigeria	-4.52 (-4.78 to -4.27)
Niue	-2.71 (-3.00 to -2.42)
North Macedonia	-10.55 (-11.01 to -10.09)
Northern Mariana Islands	-3.98 (-4.22 to -3.74)
Norway	-3.70 (-3.90 to -3.50)
Oman	-6.55 (-7.10 to -6.00)
Pakistan	-3.36 (-3.93 to -2.78)
Palau	-3.19 (-3.45 to -2.93)
Palestine	-6.38 (-6.64 to -6.12)
Panama	-6.10 (-6.41 to -5.78)
Papua New Guinea	-1.82 (-2.04 to -1.60)
Paraguay	-4.56 (-4.80 to -4.33)
Peru	-9.84 (-10.12 to -9.56)
Philippines	-3.17 (-3.40 to -2.95)
Poland	-5.53 (-5.85 to -5.21)
Portugal	-7.38 (-7.68 to -7.08)
Puerto Rico	-7.86 (-8.06 to -7.66)
Qatar	-7.39 (-7.57 to -7.21)
Republic of Korea	-10.86 (-11.18 to -10.54)
Republic of Moldova	-5.13 (-5.44 to -4.83)
Romania	-5.50 (-5.94 to -5.06)
Russian Federation	-5.06 (-5.75 to -4.37)
Rwanda	-8.64 (-9.18 to -8.09)
Saint Kitts and Nevis	-5.29 (-5.70 to -4.87)
Saint Lucia	-6.13 (-6.37 to -5.89)
Saint Vincent and the Grenadines	-6.36 (-6.67 to -6.05)
Samoa	-3.90 (-4.16 to -3.64)
San Marino	-5.86 (-7.06 to -4.65)
Sao Tome and Principe	-8.49 (-8.74 to -8.24)
Saudi Arabia	-10.62 (-10.92 to -10.31)
Senegal	-6.06 (-6.31 to -5.82)
Serbia	-10.55 (-10.81 to -10.29)
Seychelles	-2.29 (-2.63 to -1.95)
Sierra Leone	-4.63 (-4.97 to -4.29)
Singapore	-7.29 (-7.54 to -7.05)
Slovakia	-5.14 (-5.43 to -4.85)
Slovenia	-7.07 (-7.51 to -6.62)
Solomon Islands	-3.08 (-4.16 to -1.98)
Somalia	-2.41 (-3.00 to -1.82)
South Africa	-3.21 (-3.55 to -2.88)
South Sudan	-1.92 (-2.50 to -1.33)
Spain	-6.12 (-6.33 to -5.91)
Sri Lanka	-6.62 (-6.92 to -6.31)
Sudan	-7.77 (-8.11 to -7.43)

Suriname	-5.24 (-5.49 to -4.99)
Sweden	-1.57 (-1.80 to -1.34)
Switzerland	-4.10 (-4.41 to -3.79)
Syrian Arab Republic	-7.24 (-7.94 to -6.52)
Taiwan (Province of China)	-7.68 (-8.05 to -7.31)
Tajikistan	-4.42 (-4.55 to -4.30)
Thailand	-4.93 (-5.13 to -4.72)
Timor-Leste	-6.90 (-7.27 to -6.53)
Togo	-4.03 (-4.32 to -3.74)
Tokelau	-5.53 (-6.18 to -4.87)
Tonga	-3.08 (-3.48 to -2.68)
Trinidad and Tobago	-5.31 (-5.63 to -4.99)
Tunisia	-8.40 (-8.69 to -8.11)
Turkey	-12.76 (-13.15 to -12.35)
Turkmenistan	-5.57 (-5.91 to -5.23)
Tuvalu	-6.91 (-7.40 to -6.41)
Uganda	-4.88 (-5.34 to -4.42)
Ukraine	-1.47 (-2.14 to -0.79)
United Arab Emirates	-6.83 (-7.06 to -6.60)
United Kingdom	-2.06 (-2.50 to -1.61)
United Republic of Tanzania	-5.18 (-5.78 to -4.58)
United States of America	-3.32 (-3.68 to -2.96)
United States Virgin Islands	-6.50 (-6.90 to -6.11)
Uruguay	-6.27 (-6.50 to -6.05)
Uzbekistan	-4.09 (-4.42 to -3.76)
Vanuatu	-2.50 (-3.04 to -1.95)
Venezuela (Bolivarian Republic of)	-5.53 (-6.08 to -4.98)
Viet Nam	-6.80 (-7.18 to -6.43)
Yemen	-6.37 (-6.76 to -5.98)
Zambia	-7.11 (-7.73 to -6.48)
Zimbabwe	0.85 (0.52 to 1.17)
